# Elevated NR2F1 underlies the persistence of invasive disease after treatment of BRAF-mutant melanoma

**DOI:** 10.1172/JCI178446

**Published:** 2025-07-29

**Authors:** Manoela Tiago, Timothy J. Purwin, Casey D. Stefanski, Renaira Oliveira da Silva, Mitchell E. Fane, Yash Chhabra, Jelan I. Haj, Jessica L.F. Teh, Rama Kadamb, Weijia Cai, Sheera R. Rosenbaum, Vivian Chua, Nir Hacohen, Michael A. Davies, Jessie Villanueva, Inna Chervoneva, Ashani T. Weeraratna, Dan A. Erkes, Claudia Capparelli, Julio A. Aguirre-Ghiso, Andrew E. Aplin

**Affiliations:** 1 Department of Pharmacology, Physiology, and Cancer Biology, Thomas Jefferson University, Philadelphia, Pennsylvania, USA.; 2 Department of Clinical Chemistry and Toxicology, University of São Paulo, São Paulo, Brazil.; 3 Department of Cancer Signaling and Microenvironment, Fox Chase Cancer Center, Temple Health, Philadelphia, Pennsylvania, USA.; 4 Department of Cell Biology, Cancer Dormancy Institute, Montefiore Einstein Comprehensive Cancer Center, Gruss Lipper Biophotonics Center, Albert Einstein College of Medicine, Bronx, NY, USA.; 5 Department of Medical Microbiology, Immunology, and Cell Biology at SIU Medicine, Springfield, Illinois, USA.; 6 Broad Institute of MIT and Harvard, Cambridge, Massachusetts, USA.; 7 Department of Medicine, Center for Cancer Research, Massachusetts General Hospital, Boston, Massachusetts, USA.; 8 Department of Medicine, Harvard Medical School, Boston, Massachusetts, USA.; 9 Department of Melanoma Medical Oncology, The University of Texas MD Anderson Cancer Center, Houston, Texas, USA.; 10 Molecular and Cellular Oncogenesis Program and Melanoma Research Center, The Wistar Institute, Philadelphia, Pennsylvania, USA.; 11 Division of Biostatistics, Department of Pharmacology and Experimental Therapeutics and; 12 Sidney Kimmel Comprehensive Cancer Center, Thomas Jefferson University, Philadelphia, Pennsylvania, USA.; 13 Department of Biochemistry and Molecular Biology, Johns Hopkins Bloomberg School of Public Health and Department of Oncology, Sidney Kimmel Comprehensive Cancer Center, Baltimore, Maryland, USA.; 14 Department of Medical Oncology, Thomas Jefferson University, Philadelphia, Pennsylvania, USA.

**Keywords:** Oncology, Therapeutics, Drug therapy, Melanoma

## Abstract

Despite the success of targeted inhibitors in cutaneous melanoma, therapeutic responses are limited by the aged tumor microenvironment and drug-tolerant residual cells. Given the similarities between drug tolerance and cellular dormancy, we studied the dormancy marker, nuclear receptor subfamily 2 group F member 1 (NR2F1), in response to BRAF-V600E inhibitors (BRAFi) plus MEK inhibitors (MEKi) in BRAF-mutant melanoma models. Transcriptomic analysis of melanoma patient samples treated with BRAFi + MEKi showed increased NR2F1. NR2F1 was highly expressed in the drug-tolerant invasive cell state of minimal residual disease in patient-derived and mouse-derived xenografts on BRAFi + MEKi. NR2F1 over-expression was sufficient to reduce BRAFi + MEKi effects on tumor growth in vivo, and cell proliferation, death, and invasion in vitro. Effects were linked to genes involved in mTORC1 signaling. These cells were sensitive to the combination of BRAFi, MEKi plus rapamycin. Melanomas from aged mice, known to exhibit decreased responses to BRAFi + MEKi, displayed higher levels of NR2F1 compared to tumors from young mice. Depleting NR2F1 in an aged mouse melanomas improved the response to targeted therapy. These findings show high NR2F1 expression in ‘invasive-state’ residual cells and that targeting NR2F1-high cells with mTORC1 inhibitors may improve outcomes in patients with melanoma.

## Abstract 2

Clinical effects of BRAF plus MEK inhibitors are limited by drug-tolerant cells in melanoma. Studying the dormancy marker, NR2F1, in response to BRAF and MEK inhibitors reveals its role in promoting drug tolerance and highlights the use of mTORC1 inhibitors as a potential strategy for melanoma treatment.

## Graphical abstract

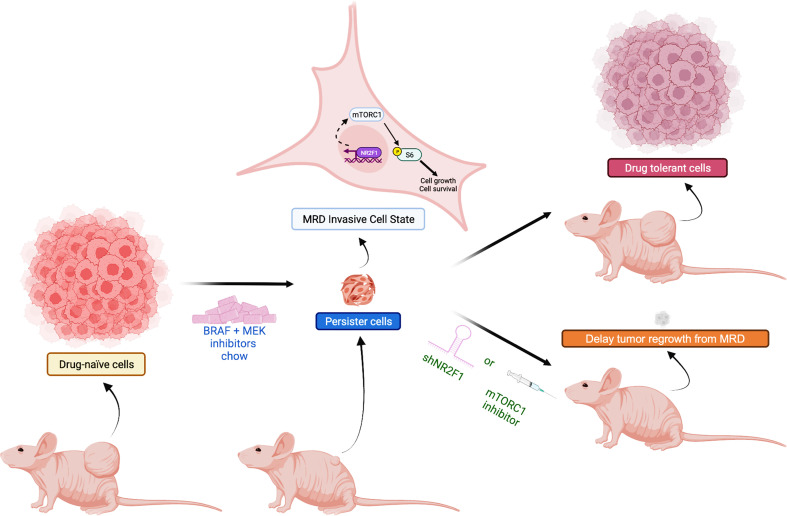

## Introduction

Current standard-of-care treatments for melanoma, including immune checkpoint inhibitors and MAPK-targeted therapies, are limited by innate and acquired resistance (1, 2). For patients with BRAF-mutant melanoma, combination treatment with BRAF inhibitors plus MEK inhibitors (BRAFi + MEKi) leads to tumor shrinkage in the majority of patients (1) but tumors progress, with a median progression-free survival (PFS) of approximately 14 months (3). Therapy response is modulated by the tumor microenvironment (TME) (4, 5). In particular, the aged TME enhances tolerance to BRAFi + MEKi by preventing tumor cell death, increasing angiogenesis, and altering normal dermal fibroblasts to secrete factors that promote resistance (6, 7). Furthermore, older mice (>52 weeks of age) exhibit lower response rates to targeted therapy relative to younger mice (8 weeks of age) (6–9). In humans, approximately 50% of melanoma cases occur in individuals over the age of 50 years, with a median age of diagnosis of approximately 63 years (6, 7).

Minimal residual disease (MRD) is characterized by slow-cycling/quiescent drug-tolerant persister (DTP) cells (10, 11) and may form a reservoir of cells that seed acquired resistance (10, 12, 13). Some subpopulations of DTP cells have invasive potential and may generate lesions at distant sites that grow out several years after treatment (14, 15). Both tumor cell dormancy and MRD following BRAFi + MEKi treatment are associated with progression (16, 17), and several drug tolerance mechanisms have been identified (12, 18). AXL overexpression and microphthalmia-associated transcription factor/SRY-related HMG-box 10 (MITF/SOX10) loss promote the survival of drug-tolerant cells (10) and are associated with transcriptional reprogramming that drives a proliferative-to-invasive phenotype switch (19, 20). Additional transcriptomic states of DTP cells exist (21–24) including a dormancy gene signature associated with activation of stress-signaling pathways such as p38 kinase and PRKR-like endoplasmic reticulum kinase (PERK) (23, 25). A better understanding of DTP cells is key for the effective prevention of acquired resistance in metastatic melanoma.

Disseminated tumor cells that have migrated to metastatic sites but remain dormant exhibit properties similar to those of DTPs (5, 26). Expression of the orphan nuclear receptor subfamily 2 group F member 1 (NR2F1, also known as COUP-TFI) promotes tumor cell dormancy via induction of SOX9/RAR/TGF/p38-driven quiescence programs in breast and prostate tumor models (27, 28); however, the role of NR2F1 in drug tolerance remains unknown. Here, we show that NR2F1 is highly expressed in melanoma patient samples characteristic of the invasive subgroup of DTPs and in cell-derived xenografts in MRD following treatment with BRAFi + MEKi. Inducible overexpression of NR2F1 reduced BRAFi + MEKi efficacy in melanoma models in vitro and in vivo. Similarly, tumors from aged mice showed higher expression of NR2F1 than did tumors from younger animals. NR2F1-expressing cells partially maintained mTORC1 signaling in the presence of BRAFi + MEKi and were sensitive to the mTORC1 inhibitor rapamycin in combination with BRAFi + MEKi. Together, our findings indicate that targeting DTP cells expressing high levels of NR2F1 may improve targeted therapy outcomes in melanoma.

## Results

### NR2F1 is upregulated in melanoma tumors following BRAFi and MEKi therapy.

**Figure 1:**
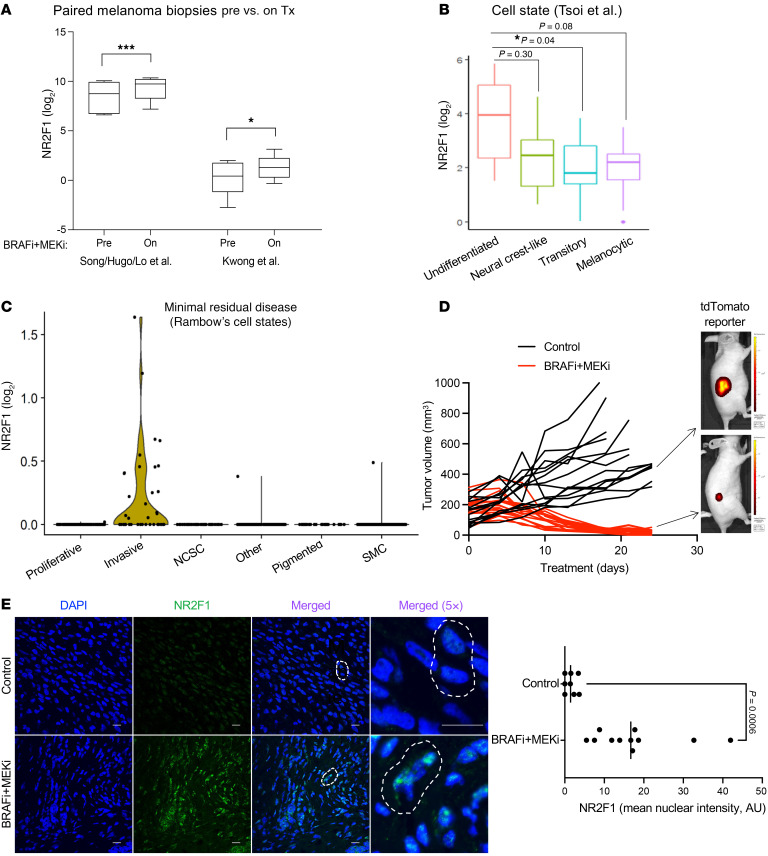
NR2F1 is highly expressed in melanoma lesions of patients on BRAFi + MEKi therapy. (**A**) Normalized expression of NR2F1 for baseline or before treatment (Pre) and early during treatment (On) tumor samples from patients who received BRAFi + MEKi combination therapy in datasets from Song et al. (32) and Kwong et al. (30). (**B**) Box plot of NR2F1 RNA-Seq gene expression data for melanoma cell lines categorized across cell states according to the dataset from Tsoi et al. (33). (**C**) Violin plot of NR2F1 expression levels by cell state in a scRNA-Seq dataset of PDX melanoma following BRAFi + MEKi treatment, according to data from Rambow et al. (12). Cell types present during MRD are shown. NCSC, neural crest stem cell; SMC, “starved-like” melanoma cell. (**D**) Tumor volume in mice bearing 1205Lu-tdTomato–labeled xenografts following continuous BRAFi + MEKi (PLX4720 200 ppm + PD0325901 7 ppm) for 3 weeks. (**E**) Representative images of detection of tdTomato fluorescence representing tumor size in xenografts after 3 weeks on BRAFi + MEKi therapy and plot showing the mean nuclear intensity of NR2F1 protein expression in 1205Lu-tdTomato cells by immunofluorescence of tumor xenografts compared with the no-drug-treatment control group. Scale bars: 100 µm. Data are presented as the mean ± SD. *P < 0.05 and ***P < 0.001, by unpaired, 2-tailed Student’s *t* test.

NR2F1 is associated with tumor dormancy in several tumor models (27, 29), and the BRAFi + MEKi drug-tolerant state exhibits traits similar to cellular dormancy, prompting us to test the link between NR2F1 and targeted therapy response. First, we analyzed published RNA-Seq datasets from patients with melanoma treated with BRAFi + MEKi (30–32). Sample expression and metadata were retrieved from the Gene Expression Omnibus (GEO) database, and analysis was limited to patients treated for up to 20 days with the combination of BRAFi (dabrafenib) and MEKi (trametinib) (Figure 1A). In the datasets from Kwong et al. (30) and Song, Hugo, Lo, and co-authors (32), *NR2F1* mRNA levels were significantly higher (*P* = 0.033 and *P* < 0.001, respectively) in BRAFi + MEKi on-treatment samples compared with patient-matched pre-treatment samples (Figure 1A). Using the Tsoi et al. melanoma drug-tolerant states dataset (33), we analyzed NR2F1 expression in the transition between phenotypic states and targeted therapy tolerance. NR2F1 expression was enhanced in the undifferentiated cell state (Figure 1B), 1of 4 melanoma states identified as highly invasive and resistant to BRAFi + MEKi. To further connect NR2F1, MRD, and therapy tolerance, we analyzed the publicly available single-cell RNA-Seq (scRNA-Seq) dataset from Rambow et al. (12). This dataset compares 4 drug-tolerant states (neural crest stem cell, invasive, “starved-like” melanoma cell, and pigmented) that are present in MRD following BRAFi + MEKi in *BRAF-*mutant melanoma patient–derived xenografts (PDXs). NR2F1 was selectively upregulated in the MRD-invasive state of DTP cells (Figure 1C and Supplemental Figure 1A; supplemental material available online with this article; https://doi.org/10.1172/JCI178446DS1).

Additionally, NR2F1 expression was assessed in the Song et al. study (32), which compares transcriptomes of patient-derived tumors on targeted therapy with MAPKi-induced cell states in human melanoma cell lines. The dynamic transcriptomic states are defined as subpopulations of DTP, DTP proliferating cells (DTP-prolif), and BRAFi/MEKi- or BRAFi/MEKi/ERKi-resistant sublines after short-term (2 days) or long-term (weeks/months) treatment (32). Using average expression data taken from this study, we observed that NR2F1 levels were increased after MAPK pathway inhibition in *BRAF*-mutant cell lines independently of their dynamic transcriptomic states (Supplemental Figures 1, B and C) (32). Together, these data suggest that NR2F1 is expressed in the tumors of patients with melanoma with MRD.

To test the association of NR2F1 with MRD in preclinical models, we identified cell lines with detectable NR2F1 expression (Supplemental Figure 1D) and treated xenografts from the NR2F1-expressing *BRAF*-mutant 1205Lu cell line with BRAFi (PLX4720) and MEKi (PD0325901). As expected, BRAFi + MEKi caused tumor regression leading to MRD by 3 weeks (Figure 1D). NR2F1 levels were enriched in MRD xenografts treated with BRAFi + MEKi compared with control tumors, as detected by immunofluorescence at the 3-week post-treatment MRD time point (Figure 1E and Supplemental Figure 1E). Additionally, we probed for the expression of NR2F1 in BRAFi + MEKi–tolerant cell lines that we have previously described (24, 34). Publicly accessible RNA-Seq analysis (24) showed increased mRNA expression of NR2F1 in two A375 BRAFi + MEKi tolerance models, CRT34 and CRT35, compared with parental A375 cells (Supplemental Figure 1F). Additionally, there was a modest but detectable increase in protein expression of NR2F1 in CRT34 and CRT35 cells cultured for 24 hours in the presence or absence of BRAFi + MEKi (Supplemental Figure 1G). Together, these data associate NR2F1 upregulation with drug tolerance and MRD following targeted therapy in cutaneous melanoma.

### NR2F1 overexpression minimizes tumor inhibition effects by BRAFi and MEKi treatment.

**Figure 2:**
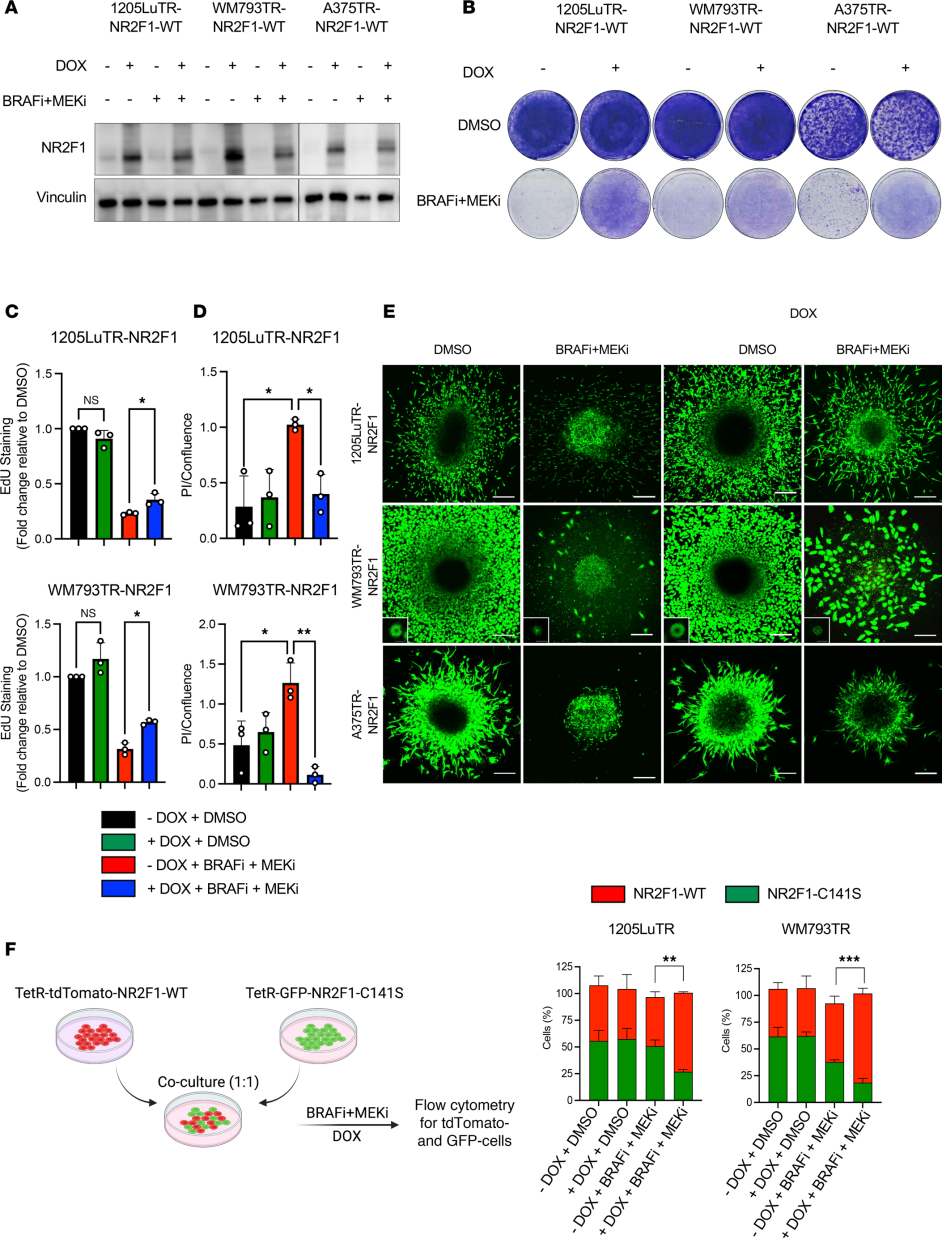
NR2F1 overexpression minimizes tumor inhibition effects by BRAFi and MEKi therapy. (**A**) NR2F1 protein levels in human *BRAF*-mutant melanoma cell lines expressing DOX-inducible NR2F1, 1205LuTR-NR2F1, WM793TR-NR2F1, and A375TR-NR2F1 after 72 hours of treatment using BRAFi + MEKi + DOX (1 μmol/L PLX4720 + 35 nmol/L PD0325901 + 100 ng/mL DOX). (**B**) Colony assay for cell lines overexpressing DOX-inducible NR2F1 after 1 week of treatment using BRAFi + MEKi + DOX. Original magnification, ×20. (**C**) Detection of S-phase cell-cycle arrest following EdU staining for the *BRAF-*mutant human melanoma cell lines listed above after 72 hours of treatment with BRAFi + MEKi + DOX. *P < 0.05, by Tukey’s test. (**D**) PI uptake over cell confluence in the *BRAF-*mutant human melanoma cell lines listed above after 72 hours of treatment with BRAFi + MEKi + DOX as determined by IncuCyte analysis. *P < 0.05 and **P < 0.01, by Tukey’s test. (**E**) Representative images of 3D tumor spheroids of the human *BRAF-*mutant melanoma cell lines 1205LuTR-NR2F1, WM793TR-NR2F1, and A375TR-NR2F1 after 72 hours of treatment with BRAFi + MEKi + DOX (1 μmol/L PLX4720 + 35 nmol/L PD0325901 + 100 ng/mL DOX). 3D tumor spheroids were stained with calcein-AM (7 μmol/L) for cell viability evaluation. Scale bars: 100 μm. (**F**) Scheme of coculturing of tdTomato cells overexpressing DOX-inducible NR2F1 WT (94) and GFP cells overexpressing a DOX-inducible dominant-negative form of NR2F1 (38) harboring a C141S point mutation within its DNA-binding domain (C141S). Cells were mixed at a ratio of 1:1 and then cocultured for 72 hours with or without BRAFi + MEKi + DOX (1 μmol/L PLX4720 + 35 nmol/L PD0325901 + 100 ng/mL DOX). Cocultures from **F** were collected and analyzed by FACS for tdTomato and GFP positivity. (**G**) The percentage of tdTomato and GFP positivity was compared with DMSO. **P < 0.01 and ***P < 0.001, by 2-way ANOVA. Data are presented as the mean ± SD.

To determine whether NR2F1 is sufficient to enhance drug tolerance, we induced the expression of NR2F1 in *BRAF*-V600E 1205Lu, WM793, and A375 melanoma cell lines and treated them with or without BRAFi + MEKi (Figure 2A). LacZ/β-gal was used as a control for the inducible protein expression system (Supplemental Figure 2A). No other members of the NR2F family were upregulated when NR2F1 was overexpressed in melanoma cell lines (Supplemental Figure 2B). In colony growth assays, inducible expression of NR2F1 did not alter melanoma cell growth in the absence of BRAFi + MEKi but was able to partially rescue colony growth in the presence of BRAFi + MEKi (Figure 2B and Supplemental Figure 2C). We further tested whether NR2F1 expression altered cell proliferation following BRAFi + MEKi by measuring S-entry phase using 5-ethynyl-2’-deoxyuridine (EdU) staining. BRAFi + MEKi–treated cells that overexpressed NR2F1 exhibited higher EdU staining than did non-NR2F1-overexpressing cells on targeted therapy (Figure 2C). Additionally, as BRAFi + MEKi treatment induces cell death (35), we tested whether NR2F1 overexpression affected cell death by measuring propidium iodide (PI) uptake via real-time IncuCyte analysis. NR2F1 overexpression significantly reduced PI uptake during BRAFi + MEKi treatment (Figure 2D).

Since drug-tolerant cells may also gain motility and interact with the extracellular matrix to promote disease progression (36, 37), we investigated how NR2F1 influences tumor invasion. Using a 3D tumor spheroid outgrowth assay*,* we found that NR2F1 expression enhanced melanoma invasiveness in spheroids treated with BRAFi + MEKi (Figure 2E and Supplemental Figure 2D). To confirm that NR2F1 confers a survival benefit to melanoma cells in the presence of BRAFi + MEKi, we inducibly expressed either tdTomato-NR2F1-WT or a dominant-negative form of NR2F1, GFP-NR2F1-C141S, in 1205Lu-TR and WM793TR melanoma cells, which express the Tet repressor (TetR). NR2F1-C141S harbors a point mutation within its DNA-binding domain (38). When cocultured at a 1:1 ratio, tdTomato-NR2F1-WT cells outgrew the GFP-NR2F1-C141S–mutant cells in the presence of BRAFi + MEKi (Figure 2, F and G). In sum, our data suggest that NR2F1 confers a pro-growth and pro-invasion state in melanoma during targeted therapy.

**Figure 3:**
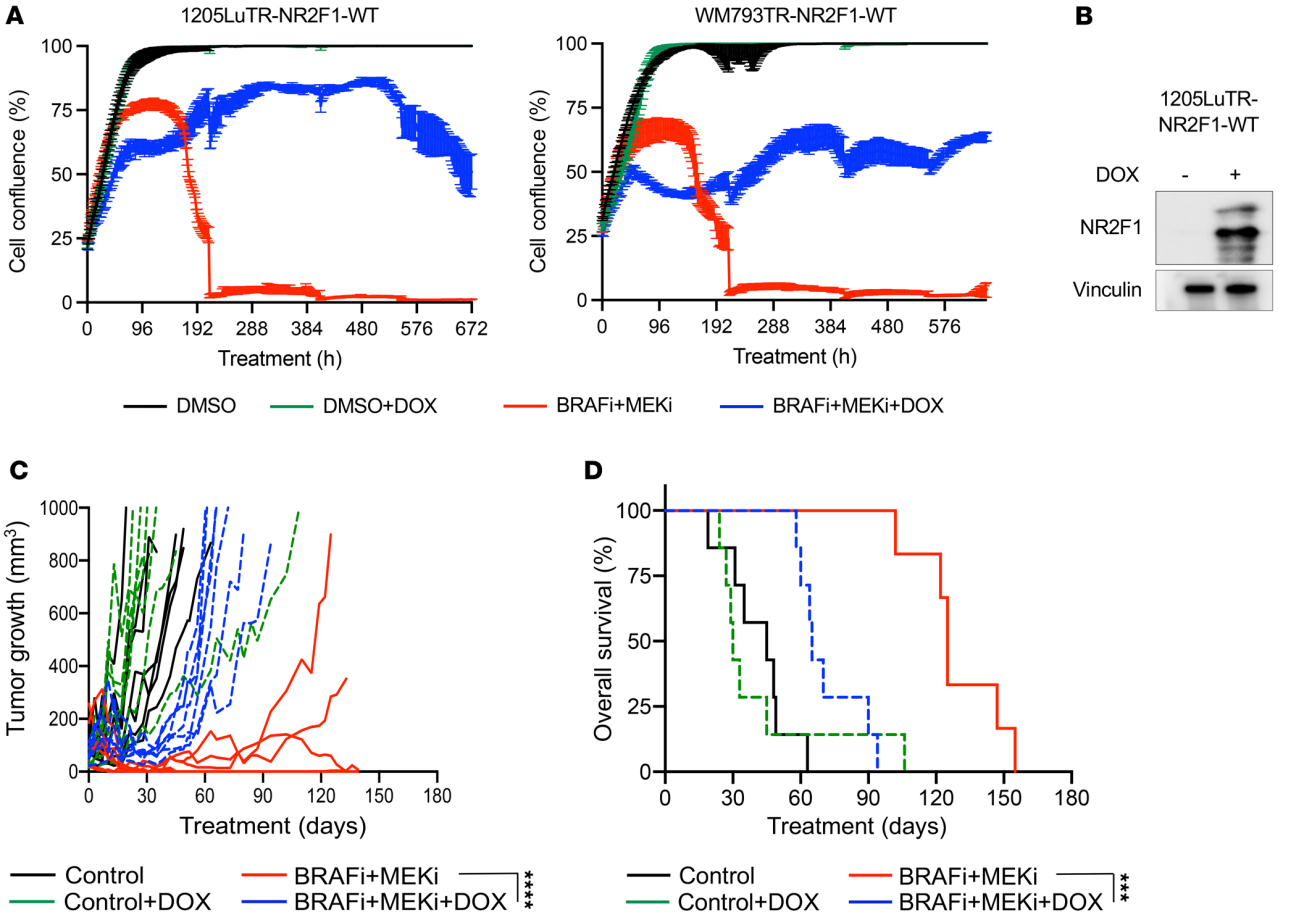
NR2F1 overexpression promotes tumor relapse following BRAF and MEK inhibitors therapy. (**A**) IncuCyte live-cell analysis for DOX-inducible cells overexpressing NR2F1 after 4 weeks of treatment using BRAFi + MEKi + DOX (1 μmol/L PLX4720 + 35 nmol/L PD0325901 + 100 ng/mL DOX). Data show the percentage of cell confluence on the plate. (**B**) NR2F1 protein levels for the DOX-inducible NR2F1-expressing cell line 1205Lu-E2F-tdTomato(tdTW)-TR-NR2F1 after 72 hours of treatment with BRAFi + MEKi + DOX. (**C**) In vivo tumor growth curves and (**D**) survival of 1205Lu xenografts with DOX-inducible NR2F1 expression following BRAFi + MEKi (200 ppm PLX4720 + 7 ppm PD0325901 + 25 mg/mL DOX) treatment. ***P < 0.001 and ****P < 0.0001, by Kaplan-Meier analysis.

Despite presenting a response evaluation criteria in solid tumors–defined (RECIST-defined) complete response to MAPK inhibitors (39), residual disease often persists, and the disease progresses in approximately 80% of patients (40–42). Thus, we tested whether NR2F1-expressing cells tolerate long periods of targeted therapy. Real-time IncuCyte monitoring of cell growth confirmed the ability of NR2F1-overexpressing cells to maintain growth following a 28-day exposure to BRAFi + MEKi across multiple melanoma lines (Figure 3A). In vivo, we observed that xenografts overexpressing NR2F1 responded to BRAFi + MEKi treatment but progressed faster over time than did noninduced tumors (Figure 3, B and C). Mice harboring tumors overexpressing NR2F1 showed poorer survival than did mice bearing control xenografts on targeted therapy (Figure 3D). Together, these data suggest that *BRAF*-mutant melanoma cells expressing high levels of NR2F1 tolerate targeted therapy and that tumors develop resistance more quickly.

### NR2F1 expression leads to enrichment of growth-related transcripts following BRAFi and MEKi therapy.

**Figure 4:**
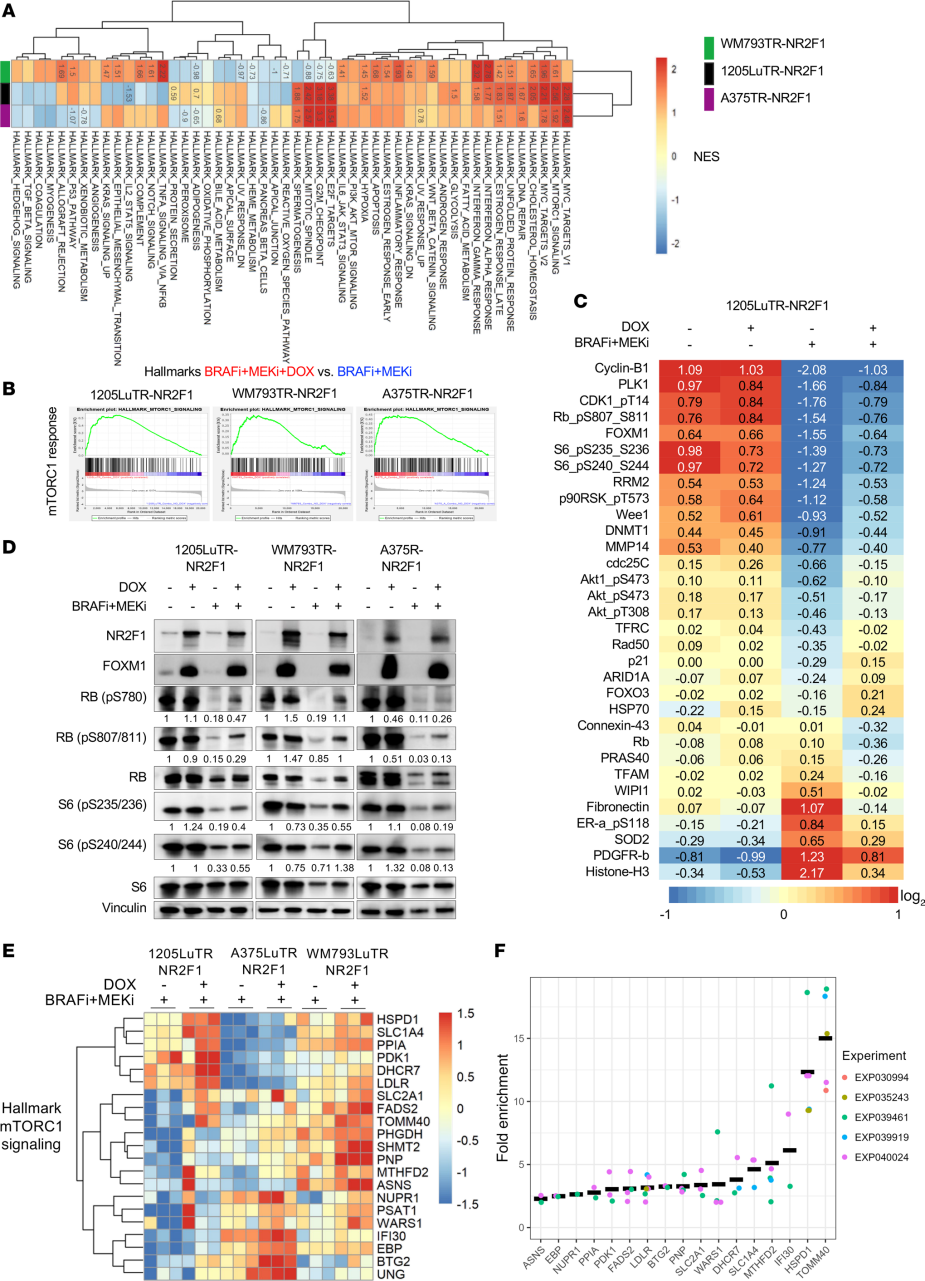
NR2F1 overexpression upregulates cell proliferation and mTORC1 signaling following BRAFi + MEKi therapy. (**A**) Heatmap showing GSEA normalized enrichment scores (NES) for the hallmark gene set collection comparing NR2F1 expression with no DOX after 72 hours of treatment with BRAFi + MEKi + DOX (1 μmol/L PLX4720 + 35 nmol/L PD0325901 + 100 ng/mL DOX) in 1205LuTR-NR2F1, WM793TR-NR2F1, and A375TR-NR2F1 cell samples. NES values are displayed for enriched gene sets using a Benjamini-Hochberg FDR (BHFDR) cutoff of 0.05. (**B**) GSEA hallmark enrichment plots showing the mTORC1 response for the same cells and drug treatments as in **A**. (**C**) RPPA analysis of the *BRAF-*mutant human melanoma cell lines 1205LuTR-NR2F1 after 72 hours of treatment with BRAFi + MEKi + DOX. Results indicate the median-centered, log_2_-transformed group average RPPA expression data for targets with at least 25% change when comparing NR2F1 expression with no DOX after 72 hours of treatment. (**D**) Representative Western blot of the RPPA-identified proteins in **C** after treatment with BRAFi + MEKi for 72 hours (*n* = 2–3). Quantification of the band densitometry (phosphorylated proteins vs. total protein [e.g., RB (pS780)/RB] normalized to no BRAFi + MEKi + no-DOX conditions] is displayed under each band. Note that the NR2F1 blot is the same here and in Supplemental Figure 4A. (**E**) Heatmap showing hierarchical clustering of commonly enriched genes in mTORC1 GSEA results for all 3 cell line comparisons of BRAFi + MEKi + DOX. (**F**) MACS2 fold enrichment values are displayed for genes with NR2F1 ChIP-Seq binding in their promoter region (*n* = 17; each ChIP-Seq experiment is represented by a dot) from the genes in **E**.

To investigate how NR2F1 may lead to targeted therapy tolerance, we performed bulk RNA-Seq and gene set enrichment analysis (GSEA) on melanoma cells with or without NR2F1 overexpression that were treated with BRAFi + MEKi. In 3 melanoma cell lines (1205Lu, WM793, and A375), NR2F1 expression induced an enrichment in the hallmark gene sets for MYC targets variant 2 and mTORC1 signaling in the presence BRAFi + MEKi compared with treated control cells that did not overexpress NR2F1 (Figure 4, A and B, and Supplemental Figure 3A). NR2F1 overexpression also increased enrichment in the hallmark gene sets for a late response to estrogen (Supplemental Figure 3B). Some pathways were only altered in 2 of the 3 cell lines. For example, enrichment hallmark gene sets for MYC targets variant 1, DNA repair, E2F targets, G_2_/M checkpoint, mitotic spindle, and spermatogenesis were upregulated in NR2F1-expressing 1205Lu and A375 cells (Supplemental Figure 3C), whereas augmentation of cholesterol homeostasis, the unfolded protein response, IFN-α response, IFN-γ response, and hypoxia were altered in 1205Lu and WM793 (Supplemental Figure 3D). Additionally, we observed an NR2F1-dependent reduction in an adipogenesis gene set in WM793 and A375 cell lines (Supplemental Figure 3E).

Given the role NR2F1 in dormant and senescent cancer cells (27, 28), we further queried these Kim et al. RNA-Seq data with dormancy “down” and “up” gene signatures (43). We found no consistently significant changes in dormancy markers (other than NR2F1) across any of the cell lines comparing BRAFi + MEKi treatment with or without NR2F1 overexpression (Supplemental Figure 3, F–H). For example, ADAM10 from the “up” signature was significantly decreased in 1205Lu and WM793 cells, but was increased in A375 cells. In the dormancy “down” signature, CDKN3, CKS2, BUB1, FOXM1, BUB1B, and TK1 were all significantly increased in 1205Lu and WM793 cells, but either were either up- or downregulated (not significantly) in A375 cells. We further probed additional dormancy markers in the data from Fane et al. (9), again finding significant changes in only 2 of 3 cell lines (for example, increased DKK1 in A375 and WM793 cells) (Supplemental Figure 3G). Next, to address the possible role of NR2F1 overexpression in senescence, we queried these RNA-Seq data with the REACTOME Cellular Senescence gene pathway, finding that only IL-6 was significantly increased in all cell lines (Supplemental Figure 3H). Together, these data suggest that NR2F1 overexpression affects multiple components of dormancy, but not always consistently between cell lines, in melanoma treated with BRAFi + MEKi.

As we observed increased S-phase entry of NR2F1-overexpressing cells during BRAFi + MEKi therapy (Figure 2C), we probed the RNA-Seq data for the Kyoto Encyclopedia of Genes and Genomes (KEGG) cell-cycle gene signature in all cell lines. GSEA analysis of the KEGG cell-cycle pathway displayed a positive enrichment of cell-cycle genes with NR2F1 overexpression in BRAFi + MEKi–treated cells (Supplemental Figure 3I). We probed for genes that were altered during NR2F1 overexpression in BRAFi + MEKi–treated cells and found that numerous genes were markedly upregulated in 2 of the 3 cells lines. For example, CDKN1A was only substantially increased in 1205Lu and WM793 cells, whereas MCM genes, CDC genes, ORC genes, BUB genes, and others were only substantially increased in 1205Lu and A375 cells (Supplemental Figure 3J). Thus, NR2F1 overexpression has a restorative effect on multiple components of the cell cycle during BRAFi + MEKi.

In parallel to RNA-Seq analyses, we tested the effect of NR2F1 on survival-related signaling pathways by reverse-phase protein array (RPPA) analysis. Consistent with the RNA-Seq and GSEA results (Figure 4, A and B, and Supplemental Figure 3), NR2F1-overexpressing melanoma cells treated with BRAFi + MEKi displayed high mTORC1 pathway readout (phosphorylated S6 [p-S6]) and higher expression of cell-cycle and survival regulators such as cyclin B1, PLK1, p-RB1, p-S6, and Wee1 than did BRAFi + MEKi–treated, non-NR2F1-overexpressing cells (Figure 4C). Altered levels of RPPA-identified proteins involved in the cell cycle and survival were validated by Western blotting and subsequent quantification (Figure 4D and Supplemental Figure 4A) (43). Overall, our data indicate that NR2F1 maintained cell growth in drug-tolerant melanoma subjected to targeted therapy, in part, through enhancement of the level of mTORC1 signaling during BRAFi + MEKi treatment.

To further assess NR2F1 modulation of mTORC1 signaling, we queried the RNA-Seq data and identified commonly enriched genes in the Hallmark mTORC1 gene set for 3 independent cell lines overexpressing NR2F1 and treated with BRAFi + MEKi. We found 21 significantly overlapping genes (Supplemental Figure 4B) involved in mTORC1 pathways, such as the *PDK1* and *HSPD1* genes, that were upregulated in the presence of NR2F1 overexpression during BRAFi + MEKi treatment (Figure 4E). We then queried publicly available MACS2 (44) NR2F1 ChIP-Seq binding data from the Gene Transcription Regulation Database (45) and found that NR2F1 bound to the promoter region of 17 of the 21 genes identified including *PDK1* and *HSPD1* (Figure 4F). Together, these data suggest that mTORC1 signaling is partially rescued after NR2F1 upregulation during BRAFi + MEKi treatment, likely occurring by NR2F1 binding to the promoter region of genes that promote mTORC1 signaling and inducing their transcription.

### Targeting mTORC1 decreases MRD during BRAFi + MEKi treatment.

**Figure 5:**
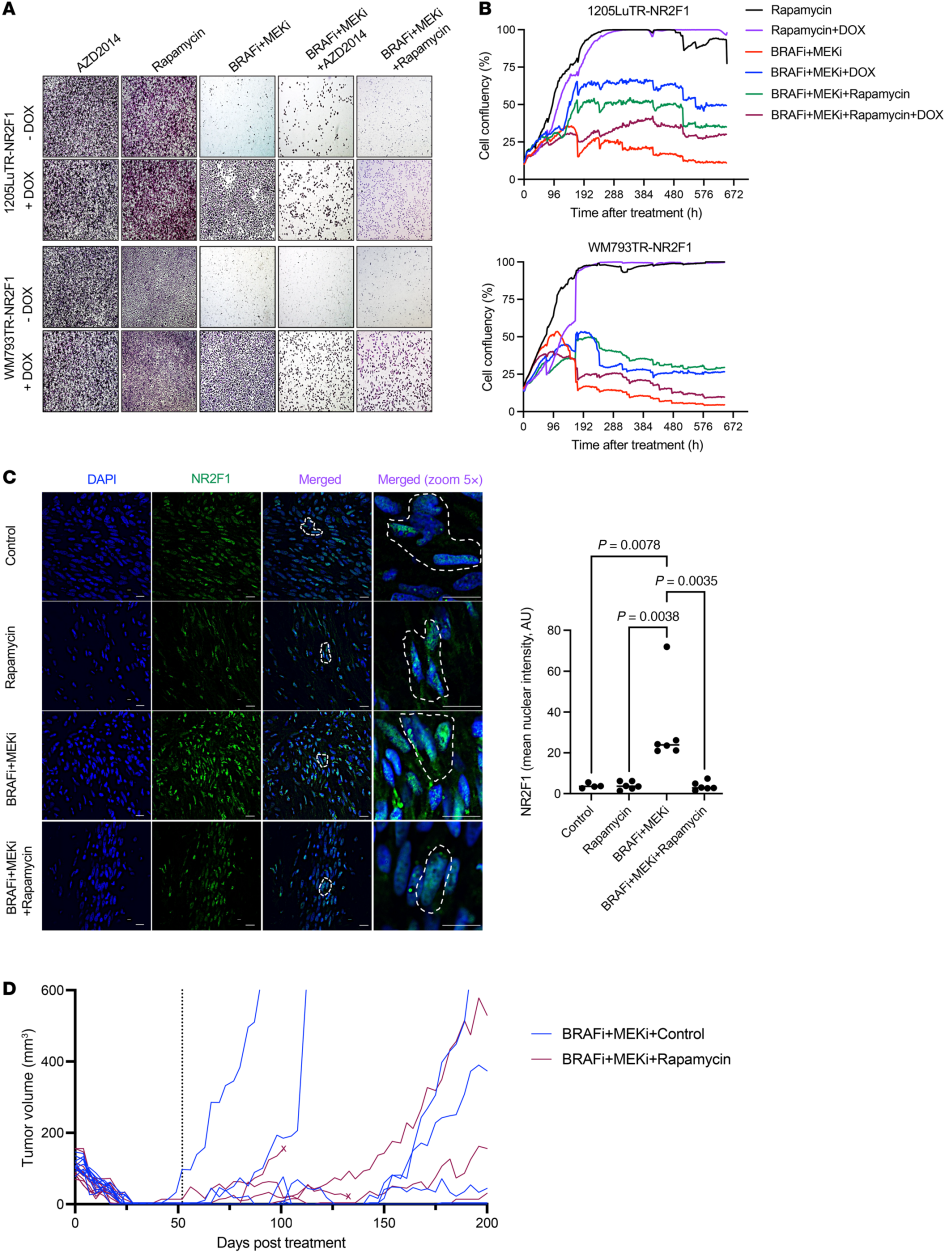
Rapamycin targets NR2F1-overexpressing drug-tolerant cells and delays tumor growth. (**A**) Colony assay for cell lines overexpressing DOX-inducible NR2F1 after 1 week of treatment with BRAFi + MEKi + mTORC1i (either 1 μmol/L AZD2014 or 1 μmol/L rapamycin). Original magnification, ×20. (**B**) IncuCyte live-cell analysis for DOX-inducible cells overexpressing NR2F1 after 4 weeks of treatment using BRAFi + MEKi + DOX. Cell growth was analyzed for percentage of cell confluence on the plate (representative of three independent experiments). (**C**) Images show the expression of NR2F1 in the MRD state following BRAFi + MEKi + rapamycin treatment (4 mg/kg) in vivo. Results are for mice bearing 1205Lu-tdTomato–labeled xenografts following continuous BRAFi + MEKi chow (200 ppm PLX4720 + 7 ppm PD0325901) for 3 weeks and then either control chow + vehicle (control), control chow + 4 mg/kg rapamycin (rapamycin), BRAFi + MEKi chow + vehicle (BRAFi + MEKi), or BRAFi + MEKi chow + rapamycin (BRAFi + MEKi + rapamycin) for 1 week. Plot on the right shows the mean nuclear intensity of NR2F1 protein expression in MRD tumor xenografts by immunofluorescence compared with the control. Statistical significance was determined by 2-way ANOVA. (**D**) Tumor volume results for mice bearing 1205Lu-tdTomato–labeled xenografts following continuous treatment with BRAFi + MEKi chow (200 ppm PLX4720 + 7 ppm PD0325901) until the tumors entered a state of MRD for several weeks (day 52 after BRAFi + MEKi), and were then given either continuous BRAFi + MEKi chow + vehicle (BRAFi + MEKi) or continuous BRAFi + MEKi chow + 4 mg/kg rapamycin twice per week (BRAFi + MEKi + rapamycin) for the duration of the experiment (treatment start indicated with dotted line on *x* axis). Tumor growth of the treated mice is shown. An “X” on a tracing denotes an animal that was euthanized for nonexperimental reasons. Data indicate the mean ± SD.

A previous study has shown that inhibition of mTORC1 signaling delays the onset of acquired resistance to BRAFi and/or MEKi (46). On the basis of the partial maintenance of mTORC1 signaling, we tested the efficacy of 2 mTOR inhibitors (AZD2014 and rapamycin) to target NR2F1-overexpressing cells (47). In NR2F1-induced cells, mTORi alone did not affect growth; however, a triple combination using BRAFi + MEKi + mTORi prevented colony formation (Figure 5A). Real-time monitoring assays of cell growth over 3 weeks indicated that NR2F1-overexpressing cells responded better to the triple combination treatment (Figure 5B), suggesting a potential therapeutic strategy to target DTP melanoma cells. Western blot analysis showed that mTORi inhibited p-S6 levels in NR2F1-overexpressing cells on BRAFi + MEKi (Supplemental Figure 5A), further supporting our proposed model, whereby DTP cells were associated with NR2F1 overexpression and persistent mTOR signaling.

We tested the effect of mTORi on residual tumors following BRAFi + MEKi in vivo. Despite using an intermittent regimen of 2 days on/5 days off AZD2014 administered with continuous BRAFi + MEKi, we had to interrupt our initial in vivo experiments because of symptoms of drug toxicity in the mice, including lethargic behavior and 20% or more body weight loss in triple combination–treated animals (Supplemental Figure 5B). To manage toxicity while retaining efficacy, we tested BRAFi + MEKi + rapamycin combination treatment for 1 week in MRD tumors. Rapamycin (sirolimus) has been shown to be well tolerated in xenograft models and in patients in clinical trials (48, 49). We found that NR2F1 levels were downregulated in MRD xenografts following BRAFi + MEKi + rapamycin compared with BRAFi + MEKi–residual tumors as detected by immunofluorescence (Figure 5C and Supplemental Figure 5C).

Given these promising results with rapamycin, we tested the effects of BRAFi + MEKi + rapamycin versus BRAFi + MEKi by treating mice with rapamycin twice a week to avoid potential toxicity. Tumors were initially treated with BRAFi + MEKi. Upon reaching MRD when tumors expressed high NR2F1 levels (Figure 1E and Figure 5C) and tumor size was stable for several weeks, rapamycin was given in combination with BRAFi + MEKi. Rapamycin delayed tumor regrowth during BRAFi + MEKi, but did not significantly affect mouse weight compared with BRAFi + MEKi alone (Figure 5D, Tables 1 and 2**,** and Supplemental Figure 5D). Specifically, for mice with tumors that regrew to 100 mm^3^, the exponential regrowth rates in each treatment group and the difference in the regrowth rates between BRAFi + MEKi + rapamycin versus BRAFi + MEKi groups were estimated (Tables 1 and 2). Tumors in BRAFi + MEKi + rapamycin group grew significantly slower (P = 0.0005) with a 3% daily increase (95% CI: 2.1%–3.9%) versus a 5.2% daily increase (95% CI: 4.4%–6.1%) in the BRAFi + MEKi group. Altogether, these data suggest that inhibiting mTORC1 with rapamycin treatment improves targeted inhibitor therapy by targeting DTP melanomas that overexpress NR2F1 and decreases regrowth rates of MRD.

### NR2F1 is overexpressed in aged BRAF-mutant melanoma models.

Age is an important poor prognostic factor in patients with melanoma (50, 51). Indeed, older patients and preclinical aged mouse models of melanoma exhibit a poor response to targeted therapy relative to younger patients/mice as a result of drug resistance mediated by the aged TME (6, 7). Aged dermal fibroblasts promote melanoma metastasis and resistance to BRAFi + MEKi therapy (6). Specifically, tumors grow more quickly and BRAFi + MEKi rapidly loses efficacy in mouse melanoma models in aged mice compared with young mice (8, 9). Since the majority of patients diagnosed with melanoma are above 66 years of age, and older individuals with melanoma respond worse to BRAFi + MEKi treatment than do younger individuals, we used aged mice to determine whether NR2F1 expression was differentially affected by an aged TME.

**Figure 6:**
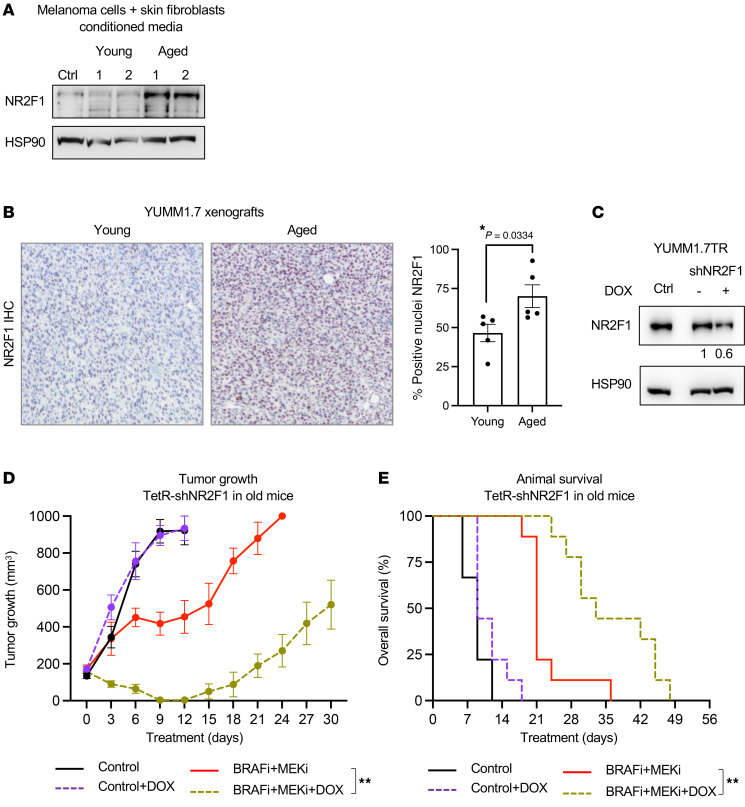
NR2F1 is overexpressed in aged *BRAF-*mutant melanoma models. (**A**) NR2F1 protein expression in cells cultured in CM derived from fibroblasts isolated from young (<35 years old) and aged (>55 years old) individuals and (**B**) YUMM1.7 tumor allografts from young versus aged mice stained for NR2F1, with quantification of the percentage of positively stained nuclei using ImageJ (NIH). Original magnification, ×100. (**C**) NR2F1 inhibition by lentiviral DOX-inducible expression of an shRNA targeting NR2F1 (tumors in both groups were >500 mm^3^ in size). (**D**) In vivo tumor growth curves (**P = 0.004127, comparing BRAFi + MEKi alone with BRAFi + MEKi + DOX). (**E**) Survival of animals with YUMM1.7 allografts with DOX-inducible shRNA targeting NR2F1 following BRAFi + MEKi + DOX (200 ppm PLX4720 + 7 ppm PD0325901 + 25 mg/mL DOX) treatment. **P = 0.004, by Kaplan-Meier analysis. Data are presented as the mean ± SD.

Melanoma cells cultured in conditioned medium (CM) derived from skin fibroblasts isolated from aged healthy human donors (>55 years old) expressed a higher level of NR2F1 compared with cells cultured in young donor (<35 years old) fibroblast–derived CM (Figure 6A). These data were supported by IHC staining findings in intradermally injected allograft primary tumors borne by aged mice (>52 weeks old) that expressed higher levels of NR2F1 compared with tumors in young mice (8 weeks old) (Figure 6B and Supplemental Figure 6); tumor sizes exceeded approximately 500 mm^3^ in size in young and aged mice (8). These data indicate that NR2F1 expression was increased in melanoma cells surrounded by an aged TME.

BRAFi + MEKi therapy is less effective in subcutaneous or intradermal tumors in an aged microenvironment (6, 8, 9). To assess the relevance of high NR2F1 expression in determining the poor response to BRAFi + MEKi in aged mice, we directly targeted NR2F1 using a tetracycline-inducible shRNA (Figure 6C). In aged mice, partial knockdown of NR2F1 elicited no effect on tumor growth in the absence of inhibitors but enhanced the effect of BRAFi + MEKi on tumor growth and delayed the time to disease progression (Figure 6D). Associated with these effects, animal survival was increased in the presence of BRAFi + MEKi treatment with NR2F1 knockdown (Figure 6E). These data suggest that NR2F1 was upregulated in tumors situated in an aged TME and that reducing NR2F1 enhanced the effects of BRAFi + MEKi treatment in an aged mouse melanoma model.

## Discussion

**Figure 7:**
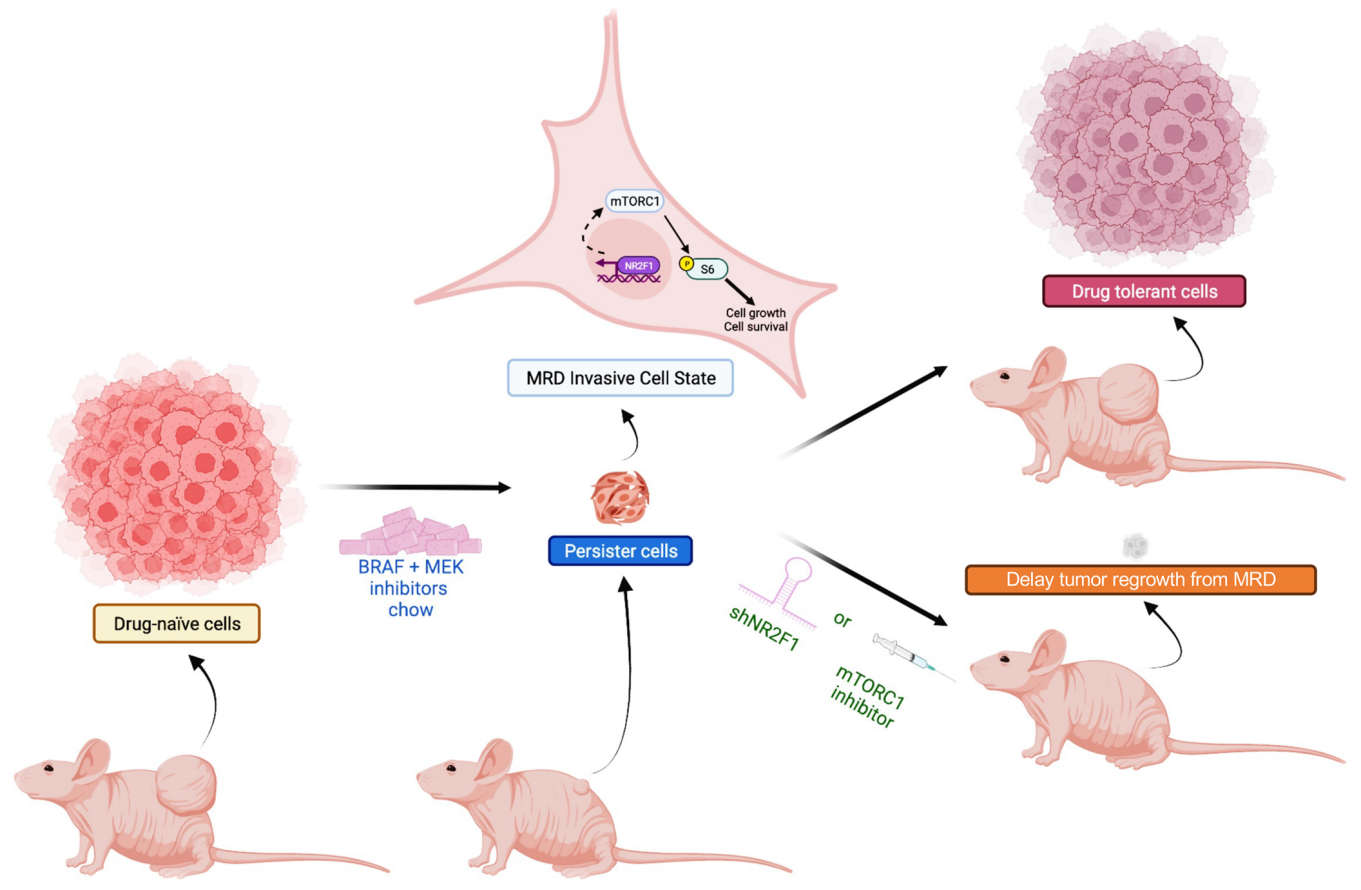
Role of NR2F1 in the persistence of MRD in melanoma. BRAFi + MEKi–tolerant persister cells express NR2F1 during MRD. NR2F1-overexpressing cells show upregulation of the mTORC1 pathway. Knockdown of NR2F1 or mTORC1 pathway inhibition delays tumor recurrence in cutaneous melanoma.

Therapeutic responses to targeted inhibitors are frequently transient, and tumors regrow from a reservoir of slow-cycling cells that survive initial treatment (52). Here, we show that NR2F1 played a role in the survival of drug-tolerant melanoma lesions. NR2F1 was upregulated in residual xenografts following BRAFi + MEKi treatment. Analysis of publicly available datasets (12, 30, 32) also revealed increased NR2F1 expression in both patient and MRD lesions from PDX samples from patients on BRAFi + MEKi treatment. NR2F1-overexpressing cells were more tolerant to BRAFi + MEKi treatment and displayed higher expression of the cell growth markers p-RB1 and p-S6. *BRAF*-mutant melanomas that emerged from an aged TME, known to be less sensitive to BRAFi + MEKi (6), also upregulated NR2F1 protein levels. Targeting BRAFi + MEKi–tolerant cells that overexpressed NR2F1 using the mTOR inhibitor rapamycin delayed melanoma regrowth and the emergence of resistance (Figure 7).

Dormancy and drug tolerance have been linked to resistance and disease relapse in melanoma (15, 16, 53–55); therefore, we questioned whether NR2F1 contributes to BRAFi + MEKi tolerance and the persistence of MRD burden. Our finding that NR2F1 was upregulated in melanoma residual tumors from patients and mice on BRAFi + MEKi treatment were consistent with studies that identified dormant marker expression in cells in the MRD state (56). Furthermore, high expression of NR2F1 is associated with tumor relapse and metastasis in salivary adenoid cystic carcinoma, urothelial cancer, and renal cancer models (57, 58). NR2F1 overexpression is reported to upregulate SOX9 through RAR-β (27); SOX9 overexpression has been linked to the promotion of migration, metastasis, and resistance to targeted therapy in melanoma (59, 60). Additional studies are warranted to determine how NR2F1 might play a role in the maintenance of drug-tolerant melanoma cells.

To investigate how NR2F1 is involved in drug-tolerant persistence, we induced overexpression of NR2F1 in melanoma cell lines. Both in vitro and in vivo results showed a higher tumor growth rate in NR2F1-overexpressing cells following BRAFi + MEKi treatment. Our transcriptomics and proteomics analyses revealed that NR2F1 overexpression led to the maintenance of mTORC1 pathway activation with BRAFi + MEKi treatment. Others have also shown that cell proliferation and PI3K/AKT/mTORC1 signaling are restored following resistance to BRAFi + MEKi therapy (61–63). NR2F1 has been previously reported to induce tumor dormancy and inhibit mTOR signaling in several cancer models (27, 29, 56, 64–67). On the other hand, high levels of NR2F1 may promote proliferation, invasion, and AKT/mTOR signaling activation via NR2F1-AS1, a long noncoding RNA with oncogenic characteristics (68, 69). NR2F1-AS1 was found to activate NR2F1 in esophageal squamous cell carcinoma to promote cancer progression by activating the hedgehog signaling pathway (68). In our study, while expression of NR2F1 alone did not inhibit cell proliferation, invasion, or mTOR signaling in our models, NR2F1 did partially rescue mTORC1 signaling in BRAF/MEK-targeted cells. Importantly, we observed that NR2F1 bound the promoter region of 17 different genes involved in mTORC1 signaling. For example, NR2F1 bound PDK1, which activates mTORC1 by phosphorylation of TSC2, thus its overexpression could upregulate mTORC1 signaling and activity (70). Furthermore, NR2F1 bound HSPD1, which can stabilize ATP5A1 in cancer cells, leading to activation of mTOR signaling (71). Future studies will determine how the binding of NR2F1 to the promoter region of various genes involved in mTORC1 signaling relates to the findings of this study.

The “invasive state” is a characteristic of dedifferentiated DTP cells emerging during targeted therapy of melanoma (21). We showed that NR2F1 expression was selectively upregulated in the invasive (12) and undifferentiated cell states (33). Furthermore, NR2F1 was sufficient to promote invasion in the presence of BRAFi + MEKi in vitro. Our data are consistent with studies showing that both the invasive and undifferentiated cell states exhibit a highly invasive phenotype and intrinsic tolerance to MAPK inhibition (21, 33, 72, 73). Others have shown that overexpression of NR2F1 increases invasiveness and metastatic potential in tumor and cancer-associated stromal cells (64, 67). For instance, salivary adenoid cystic carcinoma cells had increased expression of CXCL12 and CXCR4, enhanced invasion, and metastasis after overexpression of NR2F1; knockdown of NR2F1 reduced the invasive phenotype in these cancer cells (64). Thus, NR2F1 may modulate the invasive behavior of residual cells.

We also tested whether mTORC1 inhibitors could selectively target residual cells overexpressing NR2F1. In vitro, both the mTORC1/2i AZD2014 and the mTORC1i rapamycin, inhibited the growth of NR2F1-overexpressing cells, indicating that targeting mTORC1 may delay melanoma relapse. However, increased toxicities in mice with MRD limited the in vivo efficacy of BRAFi + MEKi + AZD2014. AZD2014 inhibits AKT activation, which may lead to increased toxicity in vivo (74). We tested a BRAFi + MEKi + rapamycin treatment regimen for better tolerability to target NR2F1-overexpressing cells. BRAFi and MEKi suppress mTOR signaling initially, but the pathway rebounds after tumors become resistant (46, 47, 49). Thus, targeting drug-tolerant cells that overexpress NR2F1 using mTORi may prevent the rise of drug-resistant subpopulations. Importantly, our findings show that a triple combination of BRAFi + MEKi + mTORi inhibited the survival of NR2F1-overexpressing cells in vivo and decreased tumor regrowth rates from MRD, suggesting that mTORi delays tumor growth of DTP cells.

Aging can negatively affect the prognosis of patients with melanoma because aged tumors are more invasive and less responsive to targeted therapy (4, 6, 7, 75). We found that NR2F1 was upregulated in the aged dermal TME compared with the younger dermal TME. We also examined the expression levels of NR2F1 in aged TME-derived melanomas and showed that knockdown of NR2F1 in tumor-bearing aged mice reduced growth and improved survival following BRAFi + MEKi. Importantly, a positive correlation exists between NR2F1 and genes associated with the “invasive” Wnt5A^hi^AXL^hi^ signature of melanoma tumors surrounded by an aged TME (9). The secretome of young and aged skin human fibroblasts has been previously defined (76), and the authors found numerous changes (90 proteins with significant up- or downregulation) related to aged fibroblasts. Retinoic acid receptor responder protein 2 (RARRES2) was increased in aged fibroblasts, and NR2F1 can be regulated by retinoic acid signaling (27). Thus, one possibility for future testing is that secreted RARRES2 affects the expression of NR2F1 in these tumors. Midkine has also been implicated in modulating NR2F1 (77), but the aforementioned secretome data show that midkine is decreased in aged fibroblasts, thus probably ruling out this mechanism. The regulation of NR2F1, especially by secreted factors, is not fully understood, and addressing this question in depth is an ongoing area of research.

Aligned with evidence that aged skin fibroblasts stimulate melanoma invasion, metastasis, and resistance to BRAFi via increased secretion of SFRP2 into the TME (4, 6, 78, 79), our data suggest that aged tumors were less likely to benefit from BRAFi + MEKi therapy, given their high levels of NR2F1, which cooperated with the aged TME to drive drug tolerance. Given the additive effects of rapamycin on BRAFi + MEKi treatment we observed in this study, treating older patients with this triple-combination therapy may circumvent NR2F1-driven BRAFi + MEKi drug tolerance in the aged TME, as long as toxicity can be avoided.

Studying drivers of MRD and drug tolerance may help identify vulnerabilities that can be exploited therapeutically in combination with BRAFi + MEKi to delay melanoma relapse. Further research is needed to confirm these findings in other cancer types upon MAPK inhibition and investigate the role of NR2F1 in immunocompetent models. Our results suggest that targeting NR2F1 in concert with BRAFi + MEKi will likely promote more durable responses to targeted therapy for BRAF-mutant melanoma in vivo.

## Methods

### Sex as a biologic variable.

For human xenograft mouse experiments, we used equal numbers of males and females to control for any sex differences. For the experiments using YUMM1.7 tumors, only male mice were used, as this is a syngeneic tumor model derived from a male mouse and thus would be rejected in female mice. More details can be found in *In vivo experiments*.

### Cell culture.

1205Lu and WM793 human melanoma cells (donated by Meenhard Herlyn, The Wistar Institute, Philadelphia, Pennsylvania, USA, in 2005) were cultured in MCDB 153 medium containing 20% Leibovitz-L15 medium, 2% FBS (Life Technologies, Thermo Fisher Scientific), 0.2% sodium bicarbonate, and 5 μg/mL insulin. A375 human melanoma cells (purchased from the American Type Culture Collection [ATCC] in 2005) were cultured in DMEM (Gibco, Life Technologies, Thermo Fisher Scientific) supplemented with 10% FBS. A375 BRAFi + MEKi tolerant cell lines, CRT34 and CRT35, were previously generated and characterized (24, 34). All cell lines used in this study were validated by Sanger sequencing as BRAF-V600 mutated. YUMM1.7 mouse melanoma cells were cultured in DMEM/F12 with 10% FBS and 1% nonessential amino acids. All cells were grown with 1% penicillin/streptomycin added to all media at 37°C in 5% CO_2_. Human cell lines were authenticated by short tandem repeat analysis. Cells were routinely assayed for mycoplasma contamination with MycoScope Kit (Genlantis).

### Inhibitors and reagents.

The inhibitors PLX4720 (BRAFi), PD0325901 (MEKi), and AZD2014 (mTORC1/2 inhibitor) were purchased from Selleck Chemicals. Doxycycline (DOX) hyclate (82D9891) was purchased from MilliporeSigma. For in vivo experiments, PLX4720 and PD0325901 were provided by Plexxikon. The published structures for the inhibitors used in this work are shown in Supplemental Figure 7.

### Lentivirus construction and transduction.

1205LuTR, WM793TR, and A375TR Tet repressor–expressing, DOX-inducible, LacZ-overexpressing cells were previously generated (80). Transgene expression was induced with DOX (100 ng/mL). Human NR2F1-WT and NR2F1-C141S were amplified from an expression plasmid and (OriGene), subcloned into pENTR/D-TOPO (Invitrogen, Thermo Fisher Scientific), and LR recombinase was used to recombine into pLenti-4/TO/V5-DEST. Expression constructs and packaging plasmids pLP1, pLP2, and pLP/VSVG were cotransfected into Lenti-X 293T cells (Takara Bio) to generate viral particles. Cells were transduced with particles for 48 hours and then selected with hygromycin, as previously described (80).

### Generation of fluorescence-labeled cells.

For tdTomato labeling, 1205LuTR and WM793TR cells were previously transduced with tdTomato fluorescent protein (81), and cells with high expression were sorted. A similar approach was followed for GFP labeling of 1205LuTR and WM793TR cells, except that the pLV-eGFP (no. 36083, Addgene) plasmid was used.

### Western blot analysis.

Protein lysates were prepared in Laemmli sample buffer, separated by SDS-PAGE, and proteins were transferred onto PVDF membranes. Immunoreactivity was detected using HRP-conjugated secondary antibodies (CalBioTech) and chemiluminescence substrate (Thermo Fisher Scientific) on a Versadoc Imaging System (Bio-Rad). The primary antibodies used were as follows: COUP-TFI/NR2F1 (no. 6364), p-RB1 (S780, no. 9307), p-RB1 (S807/811, no. 9308), RB (no. 9309), p-S6 (S235/236, no. 4857), p-S6 (S240/244, no. 2215), S6 ribosomal protein (no. 2217), FOXM1 (no. 5436), aurora A/AIK (no. 3092), PLK1 (no. 4513), Wee1 (no. 4936), and RSK1 (no. 9333) (all from Cell Signaling Technology) and vinculin (sc-73614, Santa Cruz Biotechnology).

### RPPA analysis.

Cells were seeded in 6-well plates in media with DOX for 24 hours to induce NR2F1-WT expression. The next day, cells were treated with DMSO, BRAFi + MEKi (PLX4720 1 μmol/L + PD0325901 35 nmol/L) + DOX (100 ng/mL) for an additional 72 hours. RPPA analysis was performed at the MD Anderson Functional Proteomics Core Facility (Houston, Texas, USA), and the data were used to determine antibodies significantly different between groups for each cell line treated with or without BRAFi + MEKi and with or without DOX, as previously described (82). Relative protein levels were quantified using SuperCurve fitting and normalized for protein loading. Comparison of average normalized log_2_ values was performed using the 2-sample *t* test method with 1,000 permutations and assumed unequal variance. Antibodies with a *P* value of less than 0.05 were considered significant. Statistical calculations were performed in MATLAB (version 2015b) using the mattes function. Data points are shown as averages of 3 experimental replicates.

### RNA-Seq sample preparation and analysis.

1205LuTR-NR2F1, WM793TR-NR2F1, and A375TR-NR2F1 cells were seeded in 6-well plates in regular growth media plus DOX for 24 hours to induce NR2F1-WT expression. The day after, cells were treated with DMSO (control), with or without BRAFi + MEKi (in combination, PLX4720 1 μmol/L + PD0325901 35 nmol/L), and with or without DOX (100 ng/mL) for an additional 72 hours. Total RNA was isolated using the RNeasy Plant Mini Kit (QIAGEN). Raw RNA-Seq reads were aligned to the GRCh38 human reference genome using the Star (version 2.7.0) method (83) and GENCODE (version 35) (84) annotations. RSEM (version 1.2.28) (85) was used to quantify gene- and transcript-level expression, while gene normalization and differential expression analyses were performed using DESeq2 (version 1.28.1) (86). For each cell line, a.gct file with normalized expression data was generated using the cMap package (version 1.4.0, https://github.com/cmap/cmapR) after removing genes with no expression. GSEA (version 4.1.0) (84, 87) was performed to identify significantly altered gene sets in the MSigDB Hallmark collection (version 7.2) (87). The Signal2Noise weighted enrichment statistic was performed after collapsing from Ensembl to human gene identifiers. One thousand permutations were performed using gene sets, and FDR *q* values equaling zero are reported as less than 0.001. Heatmaps were generated using the pheatmap package (version 1.0.12, https://CRAN.R-project.org/package=pheatmap). Analyses were performed in R (version 4.0.2, https://www.R-project.org/). The GEO accession number for the RNA-Seq dataset from this study is GSE228600.

### NR2F1 ChIP-Seq binding.

NR2F1 ChIP-Seq data were evaluated for potential regulation of commonly enriched mTOR genes in multiple cancer types. MACS2 (44) ChIP-Seq binding results for experiments targeting NR2F1 (*n* = 5) were obtained from the Gene Transcription Regulation Database (GTRD) (45). Gene regulation data with promoter-transcript matches were obtained from Ensembl (version 113) (88) for the human reference genome build GRCh38. Statistically significant MACS2 peaks (*P*padj < 0.05) were used for identifying overlaps between promoter regions with annotated binding to the commonly enriched mTOR genes. Overlapping regions between MACS2 peaks and Ensembl regulatory elements was conducted using the GenomicRanges (version 1.52.0) (89), rtracklayer (version1.60.1) (90), and GenomeInfoDb (version 1.36.3, https://bioconductor.org/packages/GenomeInfoDb) packages. Heatmaps were generated using the pheatmap package (version 1.0.12, https://CRAN.R-project.org/package=pheatmap). Data were analyzed in R (version 4.3.2, https://www.r-project.org) via Rstudio (version 2023.6.1, http://www.posit.co).

### Immunofluorescence assay.

Paraffin-embedded sections from human melanoma xenograft tumors were stained, as described previously (23). Briefly, samples were stained for protein expression after quenching endogenous peroxidase activity and blocked with PBS containing 3% normal goat serum. Binding of the primary antibody was carried out at 4°C overnight and detected by fluorescence-conjugated secondary antibodies (1 hour at room temperature). Paraffin-embedded sections were stained for the following antigens: COUP-TFI/NR2F1 (ab181137, Abcam) at 1:500 dilution and vinculin (sc-73614, Santa Cruz Biotechnology) at 1:200 dilution. DAPI was used for DNA staining.

### S-phase entry analysis.

Cells were cultured in 6-well plates and treated. The thymidine analog EdU was added at a final concentration of 10 μmol/L for the last 16 hours of treatment. EdU incorporation was measured using the Click-iT Plus EdU Alexa Fluor 647 Flow Cytometry Assay Kit (Thermo Fisher Scientific) and was utilized per the manufacturer’s instructions. EdU staining was analyzed on a BD FACS LSR II Flow Cytometer (BD Biosciences; Franklin Lakes, NJ) using FlowJo software (TreeStar; Ashland, OR). Data points are shown as the averages of 3 experimental replicates.

### Crystal violet assay.

Cells were seeded in 6-well plates and treated with either DMSO or BRAFi + MEKi (PLX4720 1 μmol/L + PD0325901 35 nmol/L) and with or without DOX (100 ng/mL) for 1 week. Media with drug treatments were replaced every 2 days. Cells were washed with PBS and stained with 0.2% crystal violet in 10% buffered formalin for 20 minutes. Subsequently, wells were washed and air-dried. Plates were scanned, and pictures were taken with a Nikon Eclipse Ti inverted microscope with NIS-Elements AR 3.00 software (Nikon; Melville, NY). Analysis was performed, as described previously (82).

### IncuCyte analysis.

For real-time cell confluence quantification, cells were trypsinized and plated onto a 24-well plate. Photomicrographs were taken every 2 hours using an IncuCyte Live Cell imager (Essen Biosciences). Plate confluence was measured using IncuCyte software and presented as percentages. PI uptake measurements were derived by dividing the PI^+^ area (“Surface Fit” segmentation, read capacity unit threshold = 0.75) by cellular confluence area (“AI Confluence” segmentation).

### Tumor spheroid invasion assay.

For 3D tumor spheroid formation, cells were plated in a 1.5% agar bed (w/v) for 3 days as previously described (91). Then, 3D spheroids were embedded into collagen I solution. The next day, collagen-embedded 3D tumor spheroids were treated with either DMSO or BRAFi + MEKi (PLX4720 1 μmol/L + PD0325901 35 nmol/L), with or without DOX (100 ng/mL), for 72 hours. Cells were incubated with calcein-AM solution to mark live cells. Images were obtained using a Nikon A1R confocal microscope (Nikon). The area of the invasive front was quantified using ImageJ (NIH) and normalized to the area of the spheroid.

### In vivo experiments.

For xenograft experiments, 6- to 8-week-old athymic mice (NU/J, homozygous, 20–25 g, stock no. 002019, The Jackson Laboratory) were injected with 1205LuTR-E2F-tdtW-NR2F1 human melanoma cells. For allograft experiments, YUMM1.7-TetR-shNR2F1 (2.5 × 10^5^ cells) were injected intradermally into aged male C57BL/6 mice (>52 weeks of age; Charles River Laboratories). Mice bearing 1205Lu reporter xenografts or YUMM1.7 allografts (50–100 mm^3^) were randomly divided into 4 cohorts and fed either control chow (AIN-76A diet) or chow laced with BRAFi + MEKi (PLX4720 200 ppm + PD0325901 7 ppm; Research Diets), with or without DOX (25 mg/mL in water). Treatments were determined according to previous publications (34, 82). Xenograft cohorts included 10 animals (*n* = 5 females, *n* = 5 males) per group and allograft cohorts included 10 animals (males) per group. For the triple-combination study, mice bearing xenografts were fed BRAFi + MEKi–laced chow or the BRAFi + MEKi diet along with either AZD2014 (administered by oral gavage twice daily, 2 days on/5 days off) (81) or rapamycin 4 mg/kg (administered i.p. daily). The cohorts included 6 animals (*n* = 3 females, *n* = 3 males) per group. Mice in the control and combination-chow arms received DMSO alone by oral gavage. Tumor volume measurements and animal survival were recorded every 2–3 days. Digital caliper measurements were used to calculate tumor volumes using the formula: volume = (length × width^2^)/2. When approximately 10%–15% of body weight loss was observed, diet gel 76A (ClearH2O) was administered to minimize weight loss. Animals were sacrificed when tumors exceeded 1,000 mm^3^ in size (35, 82). To investigate the effects of rapamycin in combination with BRAFi + MEKi, mice bearing 1205Lu xenografts received BRAFi + MEKi chow until tumors regressed and reached a MRD state (stagnant growth for at least 2 weeks). Treatment of BRAFi + MEKi chow + 4 mg/kg rapamycin or vehicle (administered i.p. twice weekly) was continued for the remainder of the experiment. Tumor volume and weight were measured every 3–4 days. To counteract weight loss, mice were provided diet gel 76A as a supplement. Animals were euthanized when tumors reached 700 mm^3^ in size.

Experiments using aged mice were performed at the Johns Hopkins Bloomberg School of Public Health using C57BL/6 mice after 52 weeks of age. All animals were provided ad libitum access to food and water and housed in cages of a maximum of 5 animals. Mice were housed in sterile cages within laminar flow cage racks, and all were provided enrichment, such as nest-building materials. All mice were maintained under specific pathogen–free conditions in an animal facility at the Johns Hopkins University Bloomberg School of Public Health.

### Patient RNA-Seq datasets.

For the dataset published by Song et al. (32), raw FASTQ sequencing reads for patient-derived tumor samples before treatment and early during treatment of BRAFi + MEKi were obtained from the Sequence Read Archive (SRA) (accession code SRP066571) using the SRA toolkit (version 2.10.4) (92). Reads were aligned to the GRCh38 human reference genome using the Star method (83) and GENCODE version 30 (84) annotations. RSEM (85) was used to quantify gene- and transcript-level expression. Normalization of gene expression was performed using DESeq2 (86). Box plots were generated using the ggplot2 package (version 3.3.2, https://ggplot2.tidyverse.org/). For the Kwong et al. dataset (30), RNA-Seq data were collected from European Genome-Phenome Archive (accession EGAS000010000992). Tumor samples were limited to patients treated with dabrafenib (BRAFi) and trametinib (MEKi). We modeled log-transformed NR2F1 expression levels in a linear mixed-effects (LME) model with the fixed effects of the published article source of the data (Kwong et al. [30] vs. Song et al. [ref. 32]) and time (pre–BRAFi + MEKi vs. on BRAFi + MEKi) and random effects of the patient to adjust for correlations between 2 paired observations per patient. The final model included only the published article source of the data and time as predictors of NR2F1 expression levels. The paired difference between pre–BRAFi + MEKi and on–BRAFi + MEKi was also analyzed separately for each dataset using the Wilcoxon 2-sided, 2-sample test. The analysis was performed in R.

### Xenograft and cell line RNA-Seq datasets.

For the Rambow et al. dataset (12), data on raw scRNA-Seq counts from BRAFi + MEKi–treated cutaneous melanoma xenografts resected in different response states were downloaded from the GEO database (GSE116237). Data were analyzed using the Seurat package (93). Expression levels of NR2F1 were explored on the basis of the MRD cell type calls in Rambow et al. (12). For the Tsoi et al. dataset (33), RNA-Seq data were gathered from the GEO database (GSE80829). Melanoma cell state data were gathered from Tsoi et al. (33). The independent-samples Kruskal-Wallis test was performed using the PMCMRplus package (version 1.4.1, https://CRAN.R-project.org/package=PMCMRplus). Pairwise comparisons were calculated using Dunn’s all-pairs test. Adjusted *P* values were calculated using the Bonferroni method. Box plots were generated using the ggplot2 package (version 3.1.0, https://ggplot2.tidyverse.org/). Data analyses were performed using R and RStudio (http://www.posit.co/).

### Statistics.

For in vitro studies, data are expressed as the mean ± SD and statistically analyzed using THE Student’s *t* test (2-tailed, unpaired, and assuming unequal variance). Means and SDs were calculated using at least 3 biological replicates (*n* = 3). For in vivo studies, survival curves and curves showing the percentage of tumor-free mice were analyzed using a log-rank (Mantel-Cox) test. A *P* value of less than 0.05 was considered statistically significant. All replicates for in vitro data were derived from independent experiments. No statistical method was used to predetermine sample size. No data were excluded from the analyses. Experiments using cultured cells and mice were randomized. For detection of protein levels inside the cells and interactions of proteins, Western blotting was performed and repeated twice to confirm the results.

For the in vivo rapamycin regrowth experiment (Figure 5D and Tables 1 and 2), the tumor volume measurements for each animal were interpolated using the smooth cubic splines, and resulting splines were used to approximate the time to regrow for each tumor (time to 100 mm^3^). For tumors that did not regrow to 100 mm^3^, the last day of observation was used as the time of censoring. The time to regrowth was compared between treatment groups (rapamycin vs. control) using the log-rank test. For mice with tumors that regrew to 100 mm^3^, the exponential regrowth rates were estimated for each animal by modeling log-transformed tumor volumes in a LME model with the fixed effects of day, treatment group, and interaction between day and treatment group. The model included random effects of animals in intercept and day to allow for animal-specific growth trajectories. The model was based on tumor volumes measured after tumors reached a post-treatment nadir and regrowth to at least 35 mm^3^.

### Study approval.

All xenograft experiments were reviewed and approved by the IACUC (protocol 01052) of Thomas Jefferson University and performed in a facility at Thomas Jefferson University accredited by the Association for the Assessment and Accreditation of Laboratory Animal Care (AAALAC). All mice were maintained under specific pathogen–free conditions in an AAALAC-approved Animal Facility at the Johns Hopkins University Bloomberg School of Public Health (Animal Welfare Assurance no. A3272-01). The Johns Hopkins University IACUC approved this protocol (MO22H405).

### Data and materials availability.

All data generated in this study are available within the article and the Supporting Data Values file. All raw Western blots are also included in the raw Western blot document. All RNA-Seq data are accessible on in the NCBI’s GEO database (GSE228600). All code used in this study is available at: https://github.com/AplinLabBioinformatics/Tiago_NR2F1 All unique/stable reagents generated in this study are available from the corresponding author with a completed materials transfer agreement.

## Supplementary Material

Supplemental data

Unedited blot and gel images

Supporting data values

## Figures and Tables

**Table 1:**
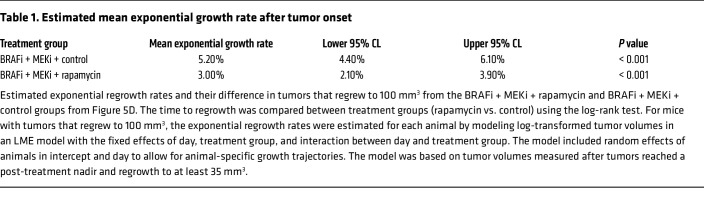
Estimated mean exponential growth rate after tumor onset

**Table 2:**
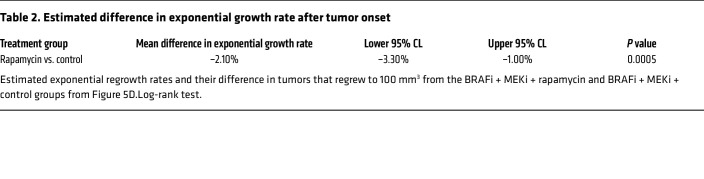
Estimated difference in exponential growth rate after tumor onset

## Version 1 07/30/2025

In-Press Preview

## Version 2 09/16/2025

Electronic publication

## References

[1] Michielin O (2020). Evolving impact of long-term survival results on metastatic melanoma treatment. J Immunother Cancer.

[2] Bhave P (2021). Melanoma recurrence patterns and management after adjuvant targeted therapy: a multicentre analysis. Br J Cancer.

[3] Savoia P (2020). Clinical implications of acquired BRAF inhibitors resistance in melanoma. Int J Mol Sci.

[4] Fane M, Weeraratna AT (2020). How the ageing microenvironment influences tumour progression. Nat Rev Cancer.

[5] Bragado P (2012). Microenvironments dictating tumor cell dormancy. Recent Results Cancer Res.

[6] Kaur A (2016). sFRP2 in the aged microenvironment drives melanoma metastasis and therapy resistance. Nature.

[7] Alicea GM (2020). Changes in aged fibroblast lipid metabolism induce age-dependent melanoma cell resistance to targeted therapy via the fatty acid transporter FATP2. Cancer Discov.

[8] Fane ME (2022). Stromal changes in the aged lung induce an emergence from melanoma dormancy. Nature.

[9] Chhabra Y (2024). Sex-dependent effects in the aged melanoma tumor microenvironment influence invasion and resistance to targeted therapy. Cell.

[10] Swayden M (2020). Tolerant/persister cancer cells and the path to resistance to targeted therapy. Cells.

[11] Atkins MB (2021). The state of melanoma: emergent challenges and opportunities. Clin Cancer Res.

[12] Rambow F (2018). Towards minimal residual disease-directed therapy in melanoma. Cell.

[13] Bristot IJ (2020). Metabolic rewiring in melanoma drug-resistant cells. Crit Rev Oncol Hematol.

[14] Ahn A (2017). The slow cycling phenotype: a growing problem for treatment resistance in melanoma. Mol Cancer Ther.

[15] Perego M (2018). A slow-cycling subpopulation of melanoma cells with highly invasive properties. Oncogene.

[16] Jahanban-Esfahlan R (2019). Tumor cell dormancy: threat or opportunity in the fight against cancer. Cancers (Basel).

[17] Risson E (2020). The current paradigm and challenges ahead for the dormancy of disseminated tumor cells. Nat Cancer.

[18] Glasheen MQ (2023). Targeting upregulated cIAP2 in SOX10-deficient drug tolerant melanoma. Mol Cancer Ther.

[19] Zhang G, Herlyn M (2014). Linking SOX10 to a slow-growth resistance phenotype. Cell Res.

[20] Uka R (2020). Temporal activation of WNT/β-catenin signaling is sufficient to inhibit SOX10 expression and block melanoma growth. Oncogene.

[21] Kemper K (2014). Phenotype switching: tumor cell plasticity as a resistance mechanism and target for therapy. Cancer Res.

[22] Yeh AC, Ramaswamy S (2015). Mechanisms of cancer cell dormancy — another hallmark of cancer?. Cancer Res.

[23] Qin Y (2021). PERK mediates resistance to BRAF inhibition in melanoma with impaired PTEN. NPJ Precis Oncol.

[24] Capparelli C (2022). Targeting SOX10-deficient cells to reduce the dormant-invasive phenotype state in melanoma. Nat Commun.

[25] Aguirre-Ghiso JA (2003). ERK(MAPK) activity as a determinant of tumor growth and dormancy; regulation by p38(SAPK). Cancer Res.

[26] Mikubo M (2021). Mechanism of drug tolerant persister cancer cells: the landscape and clinical implication for therapy. J Thorac Oncol.

[27] Sosa MS (2015). NR2F1 controls tumour cell dormancy via SOX9- and RARβ-driven quiescence programmes. Nat Commun.

[28] Borgen E (2018). NR2F1 stratifies dormant disseminated tumor cells in breast cancer patients. Breast Cancer Res.

[29] Rodriguez-Tirado C (2022). NR2F1 is a barrier to dissemination of early-stage breast cancer cells. Cancer Res.

[30] Kwong LN (2015). Co-clinical assessment identifies patterns of BRAF inhibitor resistance in melanoma. J Clin Invest.

[31] Kakavand H (2017). PD-L1 expression and immune escape in melanoma resistance to MAPK inhibitors. Clin Cancer Res.

[32] Song C (2018). Recurrent tumor cell-intrinsic and -extrinsic alterations during MAPKi-induced melanoma regression and early adaptation. Cancer Discov.

[33] Tsoi J (2018). Multi-stage differentiation defines melanoma subtypes with differential vulnerability to drug-induced iron-dependent oxidative stress. Cancer Cell.

[34] Sanchez IM (2019). In vivo ERK1/2 reporter predictively models response and resistance to combined BRAF and MEK inhibitors in melanoma. Mol Cancer Ther.

[35] Erkes DA (2020). Mutant BRAF and MEK inhibitors regulate the tumor immune microenvironment via pyroptosis. Cancer Discov.

[36] Travnickova J (2019). Zebrafish MITF-low melanoma subtype models reveal transcriptional subclusters and MITF-independent residual disease. Cancer Res.

[37] Dilshat R (2021). MITF reprograms the extracellular matrix and focal adhesion in melanoma. Elife.

[38] Adam F (2000). COUP-TFI (chicken ovalbumin upstream promoter-transcription factor I) regulates cell migration and axogenesis in differentiating P19 embryonal carcinoma cells. Mol Endocrinol.

[39] Eisenhauer EA (2009). New response evaluation criteria in solid tumours: revised RECIST guideline (version 1.1). Eur J Cancer.

[40] Robert C (2019). Five-year outcomes with dabrafenib plus trametinib in metastatic melanoma. N Engl J Med.

[41] Dummer R (2022). COLUMBUS 5-year update: a randomized, open-label, phase III trial of encorafenib plus binimetinib versus vemurafenib or encorafenib in patients with *BRAF* V600-mutant melanoma. J Clin Oncol.

[42] Ascierto PA (2020). Update on tolerability and overall survival in COLUMBUS: landmark analysis of a randomised phase 3 trial of encorafenib plus binimetinib vs vemurafenib or encorafenib in patients with BRAF V600-mutant melanoma. Eur J Cancer.

[43] Kim RS (2012). Dormancy signatures and metastasis in estrogen receptor positive and negative breast cancer. PLoS One.

[44] Zhang Y (2008). Model-based analysis of ChIP-Seq (MACS). Genome Biol.

[45] Yevshin I (2019). GTRD: a database on gene transcription regulation-2019 update. Nucleic Acids Res.

[46] Deng W (2012). Role and therapeutic potential of PI3K-mTOR signaling in de novo resistance to BRAF inhibition. Pigment Cell Melanoma Res.

[47] Gopal YN (2014). Inhibition of mTORC1/2 overcomes resistance to MAPK pathway inhibitors mediated by PGC1α and oxidative phosphorylation in melanoma. Cancer Res.

[48] Guba M (2002). Rapamycin inhibits primary and metastatic tumor growth by antiangiogenesis: involvement of vascular endothelial growth factor. Nat Med.

[49] Day TA (2019). Inhibition of mTOR signaling and clinical activity of rapamycin in head and neck cancer in a window of opportunity trial. Clin Cancer Res.

[50] Page AJ (2012). Increasing age is associated with worse prognostic factors and increased distant recurrences despite fewer sentinel lymph node positives in melanoma. Int J Surg Oncol.

[51] Balch CM (2013). Age as a prognostic factor in patients with localized melanoma and regional metastases. Ann Surg Oncol.

[52] Lo RS, Shi H (2014). Detecting mechanisms of acquired BRAF inhibitor resistance in melanoma. Methods Mol Biol.

[53] De Angelis ML (2019). Stem cell plasticity and dormancy in the development of cancer therapy resistance. Front Oncol.

[54] Cackowski FC, Heath EI (2022). Prostate cancer dormancy and recurrence. Cancer Lett.

[55] Shepherd TG, Dick FA (2022). Principles of dormancy evident in high-grade serous ovarian cancer. Cell Div.

[56] Ruth JR (2021). Cellular dormancy in minimal residual disease following targeted therapy. Breast Cancer Res.

[57] Perets R (2012). Genome-wide analysis of androgen receptor targets reveals COUP-TF1 as a novel player in human prostate cancer. PLoS One.

[58] Hao Y (2006). Gene expression profiling reveals stromal genes expressed in common between Barrett’s esophagus and adenocarcinoma. Gastroenterology.

[59] Cheng PF (2015). Methylation-dependent SOX9 expression mediates invasion in human melanoma cells and is a negative prognostic factor in advanced melanoma. Genome Biol.

[60] Yang X (2019). SOX9 is a dose-dependent metastatic fate determinant in melanoma. J Exp Clin Cancer Res.

[61] Kruiswijk F (2016). Targeted inhibition of metastatic melanoma through interference with Pin1-FOXM1 signaling. Oncogene.

[62] Irvine M (2018). Oncogenic PI3K/AKT promotes the step-wise evolution of combination BRAF/MEK inhibitor resistance in melanoma. Oncogenesis.

[63] Wang B (2021). Targeting mTOR signaling overcomes acquired resistance to combined BRAF and MEK inhibition in BRAF-mutant melanoma. Oncogene.

[64] Gao XL (2019). NR2F1 contributes to cancer cell dormancy, invasion and metastasis of salivary adenoid cystic carcinoma by activating CXCL12/CXCR4 pathway. BMC Cancer.

[65] Borriello L (2022). Primary tumor associated macrophages activate programs of invasion and dormancy in disseminating tumor cells. Nat Commun.

[66] Khalil BD (2022). An NR2F1-specific agonist suppresses metastasis by inducing cancer cell dormancy. J Exp Med.

[67] Wu R (2022). NR2F1, a tumor dormancy marker, is expressed predominantly in cancer-associated fibroblasts and is associated with suppressed breast cancer cell proliferation. Cancers (Basel).

[68] Zhang Y (2019). NR2F1-induced NR2F1-AS1 promotes esophageal squamous cell carcinoma progression via activating Hedgehog signaling pathway. Biochem Biophys Res Commun.

[69] Liu Y (2022). Hypoxia-induced long noncoding RNA NR2F1-AS1 maintains pancreatic cancer proliferation, migration, and invasion by activating the NR2F1/AKT/mTOR axis. Cell Death Dis.

[70] Castel P (2016). PDK1-SGK1 signaling sustains AKT-independent mTORC1 activation and confers resistance to PI3Kα inhibition. Cancer Cell.

[71] Zhang Y (2024). HSPD1 supports osteosarcoma progression through stabilizing ATP5A1 and thus activation of AKT/mTOR signaling. Int J Biol Sci.

[72] Muller J (2014). Low MITF/AXL ratio predicts early resistance to multiple targeted drugs in melanoma. Nat Commun.

[73] Shaffer SM (2017). Rare cell variability and drug-induced reprogramming as a mode of cancer drug resistance. Nature.

[74] Guichard SM (2015). AZD2014, an inhibitor of mTORC1 and mTORC2, is highly effective in ER+ breast cancer when administered using intermittent or continuous schedules. Mol Cancer Ther.

[75] Li CH (2022). Age influences on the molecular presentation of tumours. Nat Commun.

[76] Kaur A (2019). Remodeling of the collagen matrix in aging skin promotes melanoma metastasis and affects immune cell motility. Cancer Discov.

[77] Tang Y (2022). Midkine expression by stem-like tumor cells drives persistence to mTOR inhibition and an immune-suppressive microenvironment. Nat Commun.

[78] Kim E (2013). Senescent fibroblasts in melanoma initiation and progression: an integrated theoretical, experimental, and clinical approach. Cancer Res.

[79] Elkhattouti A (2015). Stromal fibroblast in age-related cancer: role in tumorigenesis and potential as novel therapeutic target. Front Oncol.

[80] Abel EV, Aplin AE (2010). FOXD3 is a mutant B-RAF-regulated inhibitor of G(1)-S progression in melanoma cells. Cancer Res.

[81] Teh JL (2016). An in vivo reporter to quantitatively and temporally analyze the effects of CDK4/6 inhibitor-based therapies in melanoma. Cancer Res.

[82] Tiago M (2020). Targeting BRD/BET proteins inhibits adaptive kinome upregulation and enhances the effects of BRAF/MEK inhibitors in melanoma. Br J Cancer.

[83] Dobin A (2013). STAR: ultrafast universal RNA-Seq aligner. Bioinformatics.

[84] Frankish A (2019). GENCODE reference annotation for the human and mouse genomes. Nucleic Acids Res.

[85] Li B, Dewey CN (2011). RSEM: accurate transcript quantification from RNA-Seq data with or without a reference genome. BMC Bioinformatics.

[86] Love MI (2014). Moderated estimation of fold change and dispersion for RNA-Seq data with DESeq2. Genome Biol.

[87] Subramanian A (2005). Gene set enrichment analysis: a knowledge-based approach for interpreting genome-wide expression profiles. Proc Natl Acad Sci U S A.

[88] Zerbino DR (2015). The ensembl regulatory build. Genome Biol.

[89] Lawrence M (2013). Software for computing and annotating genomic ranges. PLoS Comput Biol.

[90] Lawrence M (2009). rtracklayer: an R package for interfacing with genome browsers. Bioinformatics.

[91] Sandri S (2016). Vemurafenib resistance increases melanoma invasiveness and modulates the tumor microenvironment by MMP-2 upregulation. Pharmacol Res.

[92] Leinonen R (2011). The Sequence read archive. Nucleic Acids Res.

[93] Stuart T (2019). Comprehensive integration of single-cell data. Cell.

[94] Frederick DT (2013). BRAF inhibition is associated with enhanced melanoma antigen expression and a more favorable tumor microenvironment in patients with metastatic melanoma. Clin Cancer Res.

