# Supplementary material for: Elevated NR2F1 underlies the persistence of invasive disease after treatment of BRAF-mutant melanoma
Source: J Clin Invest. 2025 Jul 29;135(18):e178446. doi: 10.1172/JCI178446 (PMC12435839; doi:10.1172/JCI178446)
Supplement: Supplemental data [file jci-135-178446-s190.pdf]

Supplemental Figure Legends to NR2F1 underlies the persistence of 'invasive-state' residual disease after treatment of *BRAF*-mutant melanoma.

Supplemental Figure S1. NR2F1 expression in melanoma. (A) Statistical analysis of NR2F1 expression from Fig. 1A. **(B)** Normalized expression of NR2F1 for dynamic transcriptome cell states and **(C)** normalized expression of NR2F1 for MAPKi-resistant cell states from Song/Hugo/Lo et al. 2017 (1), all in *BRAF*-mutant cell lines. **(D)** NR2F1 basal expression in *BRAF*-mutant, *NRAS*-mutant, and WT *BRAF* /WT *NRAS* melanoma cell line panel. WM1366TR-NR2F1+ was used as a positive control for NR2F1 expression. **(E)** Raw images used to quantify the expression of NR2F1 in minimal residual disease (MRD) from Fig. 1E. Magnification: 15X. **(F)** mRNA expression of NR2F1 in A375, CRT34, and CRT35 from GSE180568. **(G)** Western blot for NR2F1 expression in A375, CRT34, and CRT35 treated with or without BRAFi + MEKi for 24 hours (HSP90 as loading control). Quantification of band densitometry (NR2F1:HSP90 normalized to A375 without BRAFi + MEKi) is displayed under each band.

Supplemental Figure S2. Controls and assay quantifications for Figure 2. (A) Beta-galactosidase protein levels for the human BRAF-mutant melanoma cell line control, 1205LuTR-LacZ after 72 hours of treatment using BRAFi + MEKi + DOX (PLX4720 1 μmol/L + PD0325901 35 nmol/L + doxycycline 100 ng/mL). Vinculin was used as a loading control. **(B)** RNA-seq reads for NR2F family member (NR2F1, NR2F2, long noncoding RNA NR2F1-AS1, long noncoding RNA NR2F2-AS1, and NR2F6) expression levels for NR2F1-expressing cell lines, 1205LuTR-NR2F1 (1205Lu), A375TR-NR2F1 (A375), and WM793TR-NR2F1 (WM793) after 72 hours of treatment using BRAFi + MEKi + DOX (PLX4720 1 μmol/L + PD0325901 35 nmol/L + doxycycline 100 ng/mL). **(C)** Bar graph of colony formation assay results by percentage of the area covered by adherent cells. The bars represent the normalized values to DMSO groups. The data are shown as the mean ± SEM with *p<0.05, **p<0.01 and ****p<0.0001. **(D)** Spheroid outgrowth invasion

area quantification from Fig. 2D. Unpaired t-test *p<0.05 and **p<0.01.

Supplemental Figure S3. GSEA hallmark enrichment plots for NR2F1-overpressing melanoma cells. (A) GSEA hallmark enrichment plots for MYC1 targets variant 2; **(B)** estrogen late response genes; **(C)** MYC1 targets variant 1, DNA repair, E2F targets, G2/M checkpoint, mitotic spindle, spermatogenesis; **(D)** cholesterol homeostasis, unfolded protein response, interferon-alpha response, interferon-gamma response, hypoxia; and **(E)** adipogenesis comparing NR2F1 expression (DOX) to no DOX after 72 hours of treatment using BRAFi + MEKi + DOX (PLX4720 1 μmol/L + PD0325901 35 nmol/L + doxycycline 100 ng/mL) in melanoma cells with inducible NR2F1 overexpression. **(F)** Heatmaps showing fold change values for altered genes in Dormancy Up and Down gene sets from Kim et al. (2). **(G)** Heatmap showing fold change values for altered dormancy related genes from Fane et al. (3). **(H)** Heatmap showing fold change values of REACTOME Cellular Senescence pathway genes that were significantly altered in at least one comparison. **(I)** Enrichment plots for KEGG cell cycle gene set. **(J)** Heatmap showing fold change values of genes from KEGG cell cycle gene set that were significantly altered in at least one comparison.

Supplemental Figure S4. MAPK and cell-cycle targets in NR2F1-overexpressing melanoma cells. (A) Western blot for cell survival-related proteins in NR2F1-overexpressing cells after 72-hour treatment with BRAFi + MEKi + DOX (PLX4720 1 μmol/L + PD0325901 35 nmol/L + doxycycline 100 ng/mL). Vinculin was used as a loading control. Please note that the NR2F1 blot is the same here and in Figure 4D. **(B)** Venn diagram of the 21 significantly overlapping mTORC1-related genes modulated by NR2F1 overexpression during BRAFi + MEKi therapy, taken from RNA-seq, in A375-NR2F1, 1205Lu-NR2F1, and WM793-NR2F1.

Supplemental Figure S5. Targeting mTORC1 in NR2F1-overexpressing melanoma. (A) Western blot for phospho-S6 levels in NR2F1-overexpressing cells after 72-hour treatment with BRAFi (PLX4720 μmol/L) + MEKi (PD0325901 35 nmol/L) + AZD2014 (1 μmol/L) + DOX (doxycycline 100 ng/mL). Vinculin was used as a loading control. **(B)** 1205Lu xenograft mice weight loss for BRAFi + MEKi chow (PLX4720 200 ppm + PD0325901 7 ppm) -/+ AZD2014 (20 mg/kg) treatment groups. AZD2014 was administered by oral gavage bi-daily for 2 days on/5 days off. **(C)** Raw images used to quantify the expression of NR2F1 in MRD from Fig. 5C. Magnification: 15X. **(D)** Weight of mice treated with BRAFi + MEKi + rapamycin from Fig. 5D-E.

Supplemental Figure S6. NR2F1 expression in aged *BRAF*-mutant melanoma models. (A) Western blot for NR2F1 protein expression in YUMM1.7 allografts from young and aged mice. **(B)** Raw images used to quantify the expression of NR2F1 from Fig. 6B. Low Magnification (Mag.): 4X. High Mag.: 40X.

Supplemental Figure S7. Structures for inhibitors used in this manuscript. Source: https://pubchem.ncbi.nlm.nih.gov/

Supplemental References

1. Song C, Piva M, Sun L, Hong A, Moriceau G, Kong X, et al. Recurrent tumor cell-intrinsic and -extrinsic alterations during MAPKi-induced melanoma regression and early adaptation. Cancer Disc.. 2017:CD-17-0401.
2. Kim RS, Avivar-Valderas A, Estrada Y, Bragado P, Sosa MS, Aguirre-Ghiso JA, Segall JE. Dormancy signatures and metastasis in estrogen receptor positive and negative breast cancer. PLoS One. 2012;7(4):e35569.
3. Fane ME, Chhabra Y, Alicea GM, Maranto DA, Douglass SM, Webster MR, et al. Stromal changes in the aged lung induce an emergence from melanoma dormancy. Nature. 2022;606(7913):396-405.

A

Statistical analysis for Fig 1A:

gene_ID	gene_name	avg_log2FC	p_val	p_val_adj	CellType_numerator	CellType_denominator
ENSG00000175745	NR2F1	3.91E-01	2.14E-05	8.01E-01	invasive	pigmented
ENSG00000175745	NR2F1	3.91E-01	2.14E-05	1.53E-01	invasive	immune
ENSG00000175745	NR2F1	3.91E-01	4.79E-07	1.79E-02	invasive	NCSC
ENSG00000175745	NR2F1	3.90E-01	1.61E-12	6.05E-08	invasive	proliferative
ENSG00000175745	NR2F1	3.86E-01	2.28E-17	8.54E-13	invasive	other
ENSG00000175745	NR2F1	3.87E-01	2.58E-24	9.67E-20	invasive	SMC

B

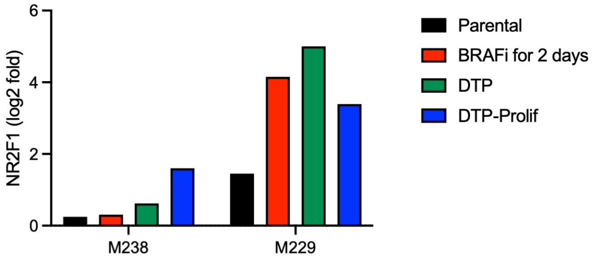

C

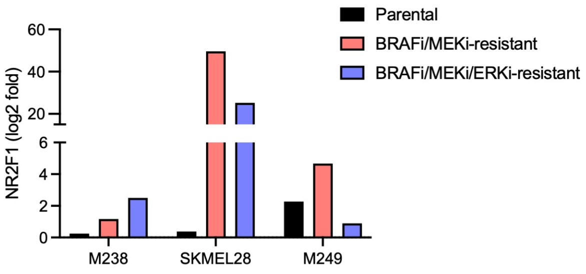

D

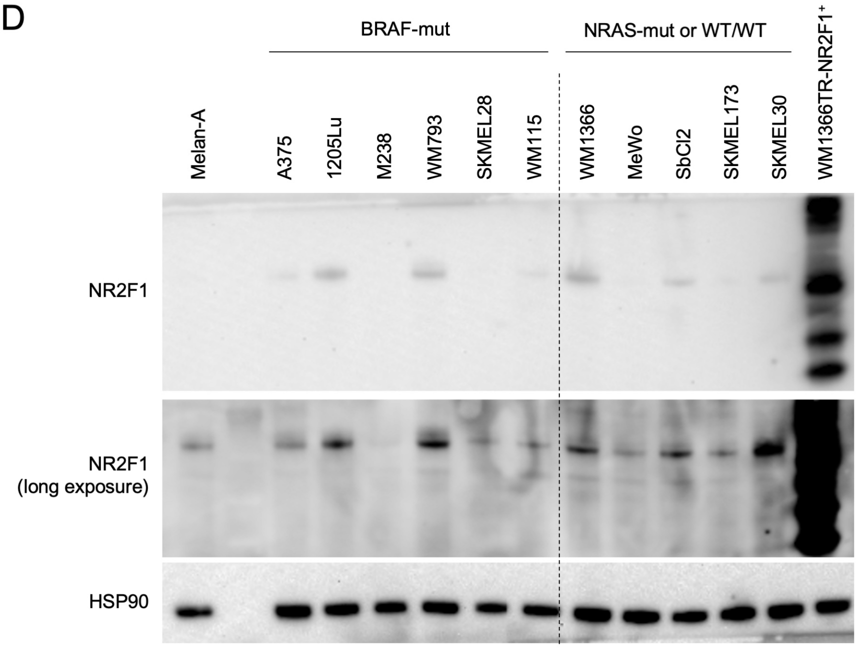

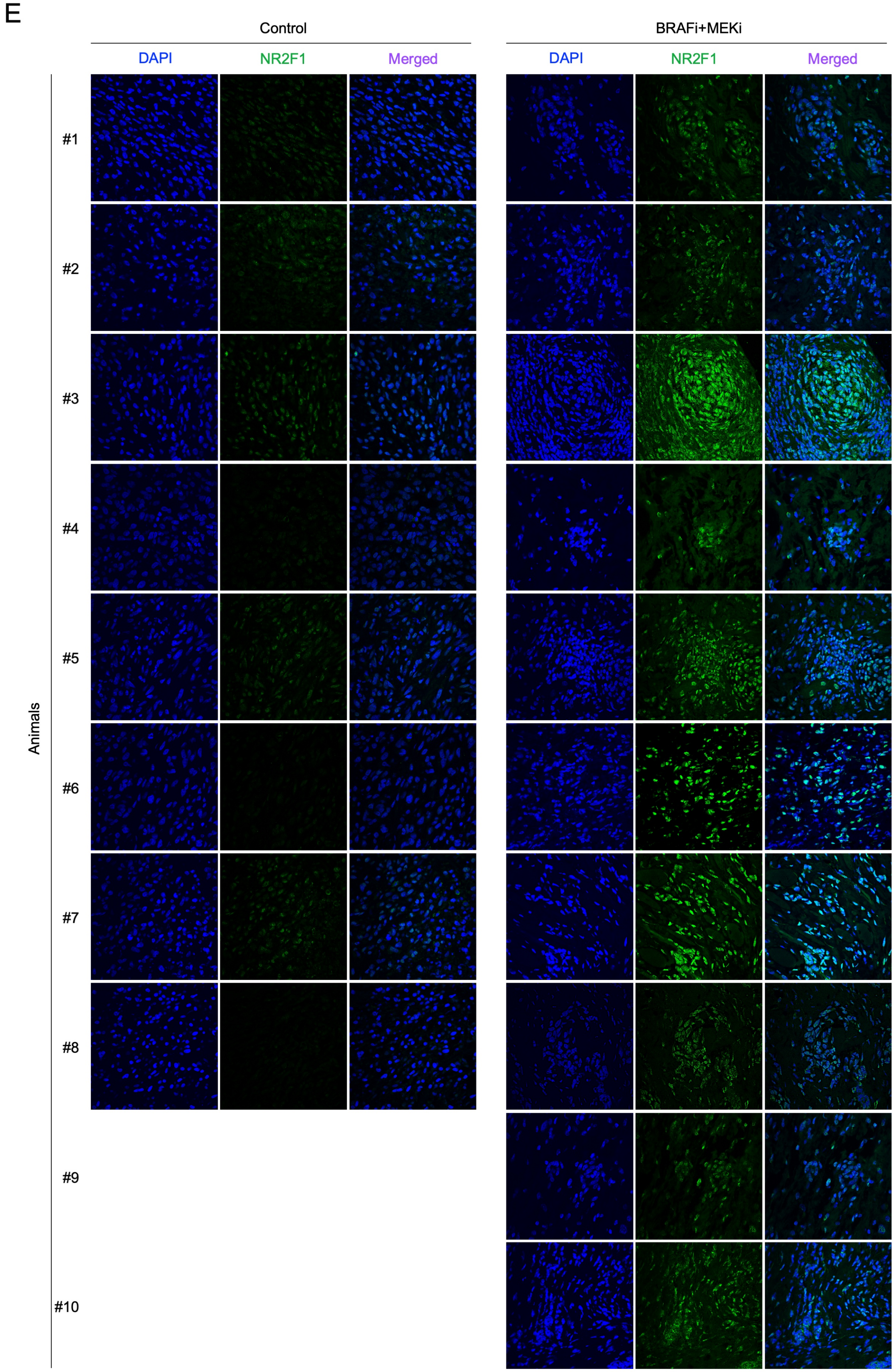
E
Control
BRAFi+MEKi
DAPI
NR2F1
Merged
DAPI
NR2F1
Merged
Animals
#1
#2
#3
#4
#5
#6
#7
#8
#9
#10

F

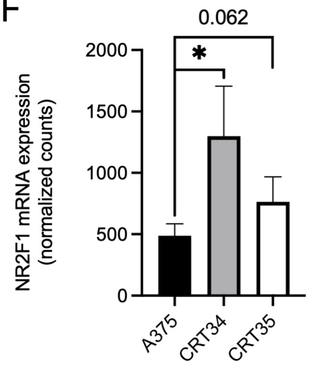
0.062
*
NR2F1 mRNA expression
(normalized counts)
2000
1500
1000
500
0
A375
CRT34
CRT35

G

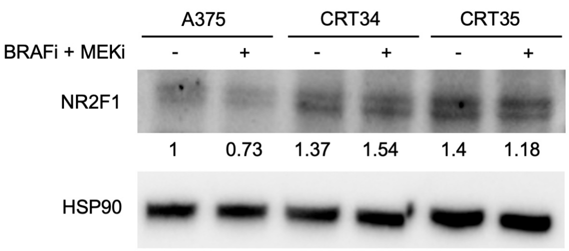
A375
CRT34
CRT35
BRAFi + MEKi
- + - + - +
NR2F1
1 0.73 1.37 1.54 1.4 1.18
HSP90

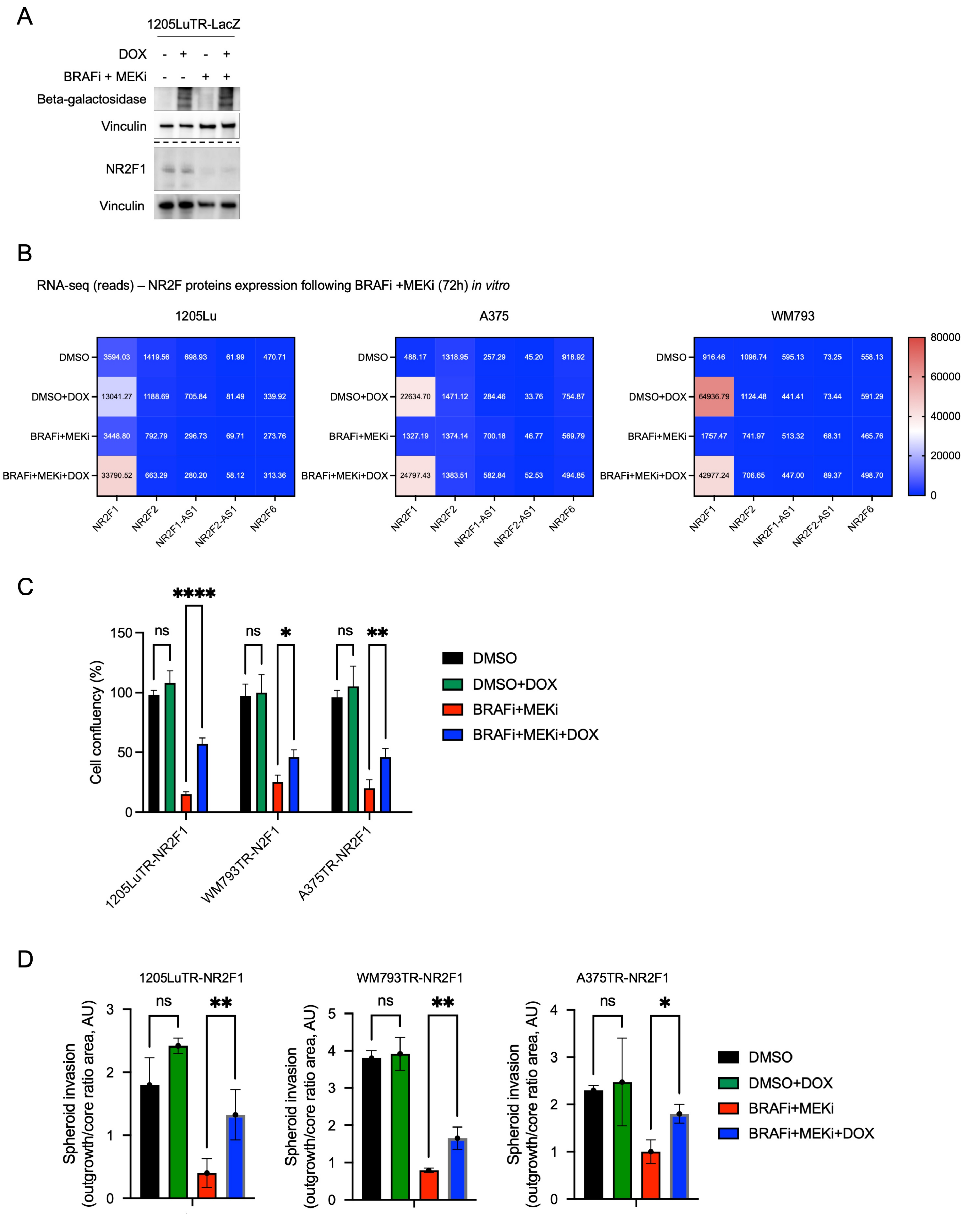
A
1205LuTR-LacZ
DOX - + - +
BRAFi + MEKi - - + +
Beta-galactosidase
Vinculin
NR2F1
Vinculin
B
RNA-seq (reads) – NR2F proteins expression following BRAFi +MEKi (72h) in vitro
1205Lu
DMSO 3594.03 1419.56 698.93 61.99 470.71
DMSO+DOX 13041.27 1188.69 705.84 81.49 339.92
BRAFi+MEKi 3448.80 792.79 296.73 69.71 273.76
BRAFi+MEKi+DOX 33790.52 663.29 280.20 58.12 313.36
A375
DMSO 488.17 1318.95 257.29 45.20 918.92
DMSO+DOX 22634.70 1471.12 284.46 33.76 754.87
BRAFi+MEKi 1327.19 1374.14 700.18 46.77 569.79
BRAFi+MEKi+DOX 24797.43 1383.51 582.84 52.53 494.85
WM793
DMSO 916.46 1096.74 595.13 73.25 558.13
DMSO+DOX 64936.79 1124.48 441.41 73.44 591.29
BRAFi+MEKi 1757.47 741.97 513.32 68.31 465.76
BRAFi+MEKi+DOX 42977.24 706.65 447.00 89.37 498.70
NR2F1 NR2F2 NR2F1-AS1 NR2F2-AS1 NR2F6
80000
60000
40000
20000
0
C
Cell confluency (%)
150
100
50
0
ns
****
ns
*
ns
**
1205LuTR-NR2F1
WM793TR-N2F1
A375TR-NR2F1
DMSO
DMSO+DOX
BRAFi+MEKi
BRAFi+MEKi+DOX
D
1205LuTR-NR2F1
WM793TR-NR2F1
A375TR-NR2F1
Spheroid invasion (outgrowth/core ratio area, AU)
ns
**
ns
**
ns
*
DMSO
DMSO+DOX
BRAFi+MEKi
BRAFi+MEKi+DOX

Tiago et al., Supplemental Figure S3

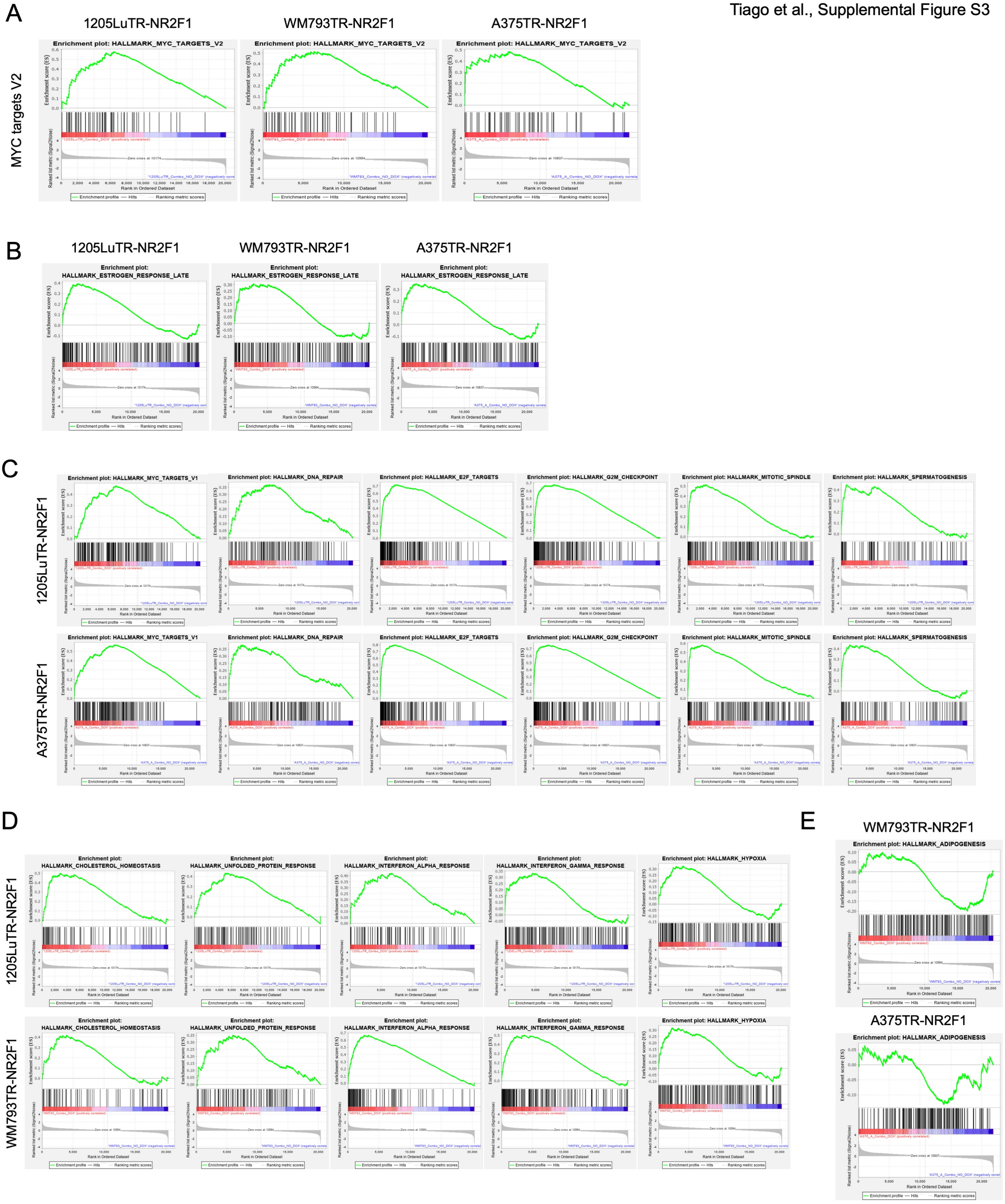

F

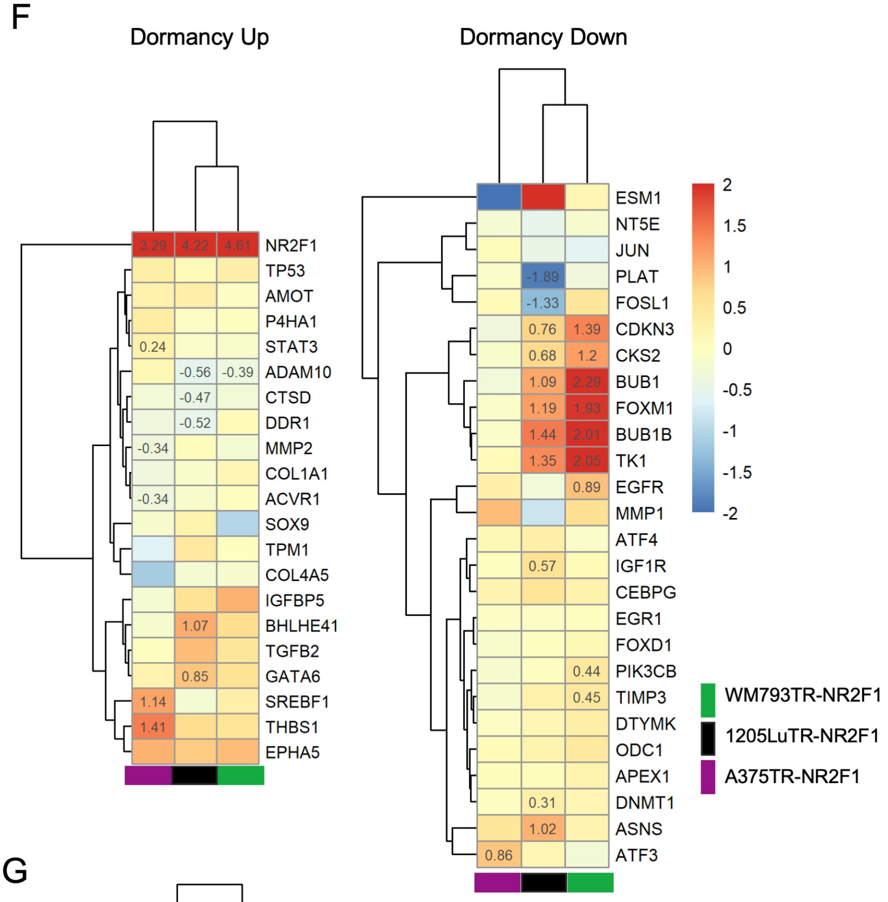
Dormancy Up
NR2F1
TP53
AMOT
P4HA1
STAT3
ADAM10
CTSD
DDR1
MMP2
COL1A1
ACVR1
SOX9
TPM1
COL4A5
IGFBP5
BHLHE41
TGFB2
GATA6
SREBF1
THBS1
EPHA5
Dormancy Down
ESM1
NT5E
JUN
PLAT
FOSL1
CDKN3
CKS2
BUB1
FOXM1
BUB1B
TK1
EGFR
MMP1
ATF4
IGF1R
CEBPG
EGR1
FOXD1
PIK3CB
TIMP3
DTYMK
ODC1
APEX1
DNMT1
ASNS
ATF3
2
1.5
1
0.5
0
-0.5
-1
-1.5
-2
WM793TR-NR2F1
1205LuTR-NR2F1
A375TR-NR2F1

G

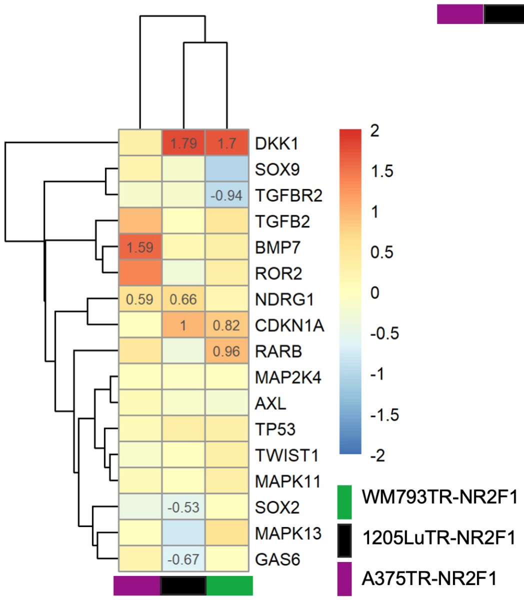
DKK1
SOX9
TGFBR2
TGFB2
BMP7
ROR2
NDRG1
CDKN1A
RARB
MAP2K4
AXL
TP53
TWIST1
MAPK11
SOX2
MAPK13
GAS6
2
1.5
1
0.5
0
-0.5
-1
-1.5
-2
WM793TR-NR2F1
1205LuTR-NR2F1
A375TR-NR2F1

H

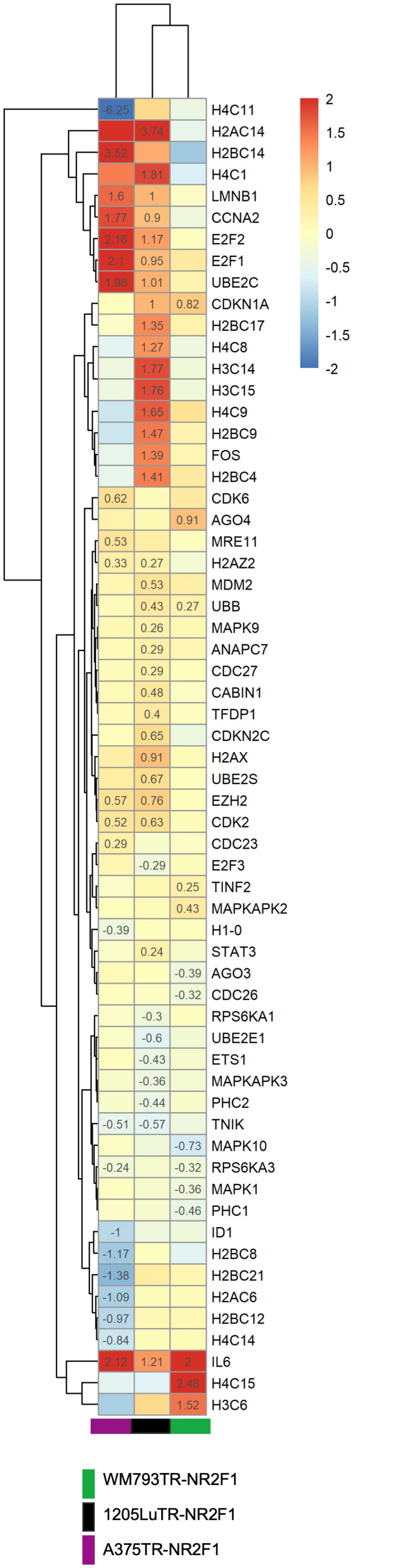
H4C11
H2AC14
H2BC14
H4C1
LMNB1
CCNA2
E2F2
E2F1
UBE2C
CDKN1A
H2BC17
H4C8
H3C14
H3C15
H4C9
H2BC9
FOS
H2BC4
CDK6
AGO4
MRE11
H2AZ2
MDM2
UBB
MAPK9
ANAPC7
CDC27
CABIN1
TFDP1
CDKN2C
H2AX
UBE2S
EZH2
CDK2
CDC23
E2F3
TINF2
MAPKAPK2
H1-0
STAT3
AGO3
CDC26
RPS6KA1
UBE2E1
ETS1
MAPKAPK3
PHC2
TNIK
MAPK10
RPS6KA3
MAPK1
PHC1
ID1
H2BC8
H2BC21
H2AC6
H2BC12
H4C14
IL6
H4C15
H3C6
2
1.5
1
0.5
0
-0.5
-1
-1.5
-2
WM793TR-NR2F1
1205LuTR-NR2F1
A375TR-NR2F1

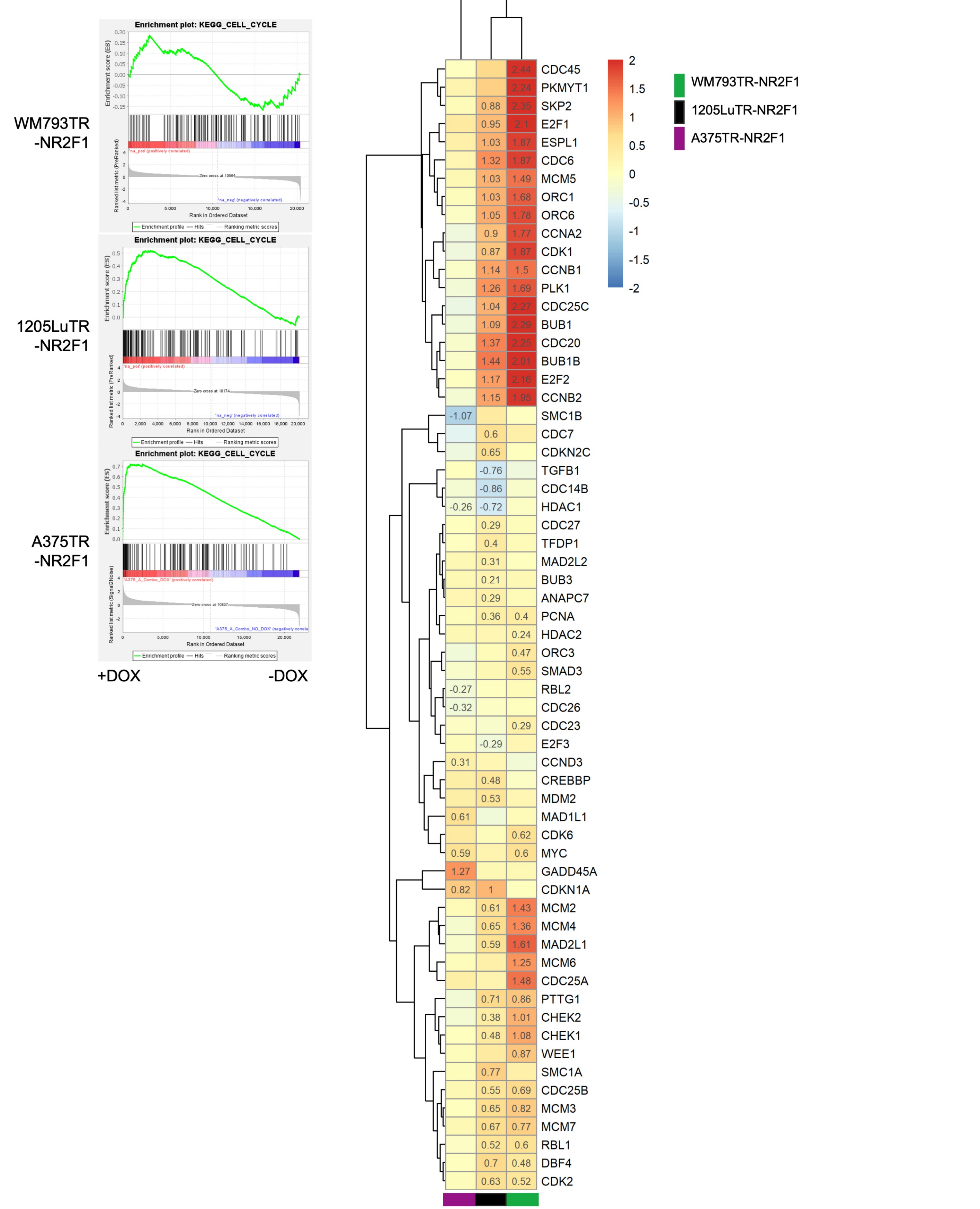
I
Enrichment plot: KEGG_CELL_CYCLE
WM793TR
-NR2F1
1205LuTR
-NR2F1
A375TR
-NR2F1
Enrichment score (ES)
Rank in Ordered Dataset
Enrichment profile — Hits — Ranking metric scores
+DOX
-DOX
J
2
1.5
1
0.5
0
-0.5
-1
-1.5
-2
WM793TR-NR2F1
1205LuTR-NR2F1
A375TR-NR2F1
2.44 CDC45
2.24 PKMYT1
0.88 2.35 SKP2
0.95 2.1 E2F1
1.03 1.87 ESPL1
1.32 1.87 CDC6
1.03 1.49 MCM5
1.03 1.68 ORC1
1.05 1.78 ORC6
0.9 1.77 CCNA2
0.87 1.87 CDK1
1.14 1.5 CCNB1
1.26 1.69 PLK1
1.04 2.27 CDC25C
1.09 2.29 BUB1
1.37 2.25 CDC20
1.44 2.01 BUB1B
1.17 2.16 E2F2
1.15 1.95 CCNB2
-1.07 SMC1B
0.6 CDC7
0.65 CDKN2C
-0.76 TGFB1
-0.86 CDC14B
-0.26 -0.72 HDAC1
0.29 CDC27
0.4 TFDP1
0.31 MAD2L2
0.21 BUB3
0.29 ANAPC7
0.36 0.4 PCNA
0.24 HDAC2
0.47 ORC3
0.55 SMAD3
-0.27 RBL2
-0.32 CDC26
0.29 CDC23
-0.29 E2F3
0.31 CCND3
0.48 CREBBP
0.53 MDM2
0.61 MAD1L1
0.62 CDK6
0.59 0.6 MYC
1.27 GADD45A
0.82 1 CDKN1A
0.61 1.43 MCM2
0.65 1.36 MCM4
0.59 1.61 MAD2L1
1.25 MCM6
1.48 CDC25A
0.71 0.86 PTTG1
0.38 1.01 CHEK2
0.48 1.08 CHEK1
0.87 WEE1
0.77 SMC1A
0.55 0.69 CDC25B
0.65 0.82 MCM3
0.67 0.77 MCM7
0.52 0.6 RBL1
0.7 0.48 DBF4
0.63 0.52 CDK2

A

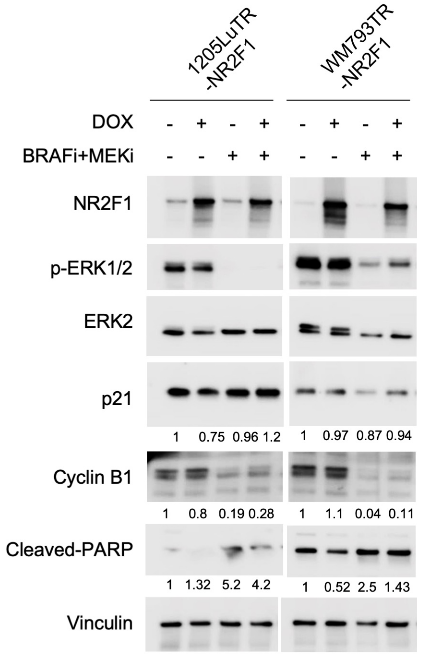
1205LuTR
-NR2F1
WM793TR
-NR2F1
DOX
BRAFi+MEKi
NR2F1
p-ERK1/2
ERK2
p21
1 0.75 0.96 1.2
1 0.97 0.87 0.94
Cyclin B1
1 0.8 0.19 0.28
1 1.1 0.04 0.11
Cleaved-PARP
1 1.32 5.2 4.2
1 0.52 2.5 1.43
Vinculin

B

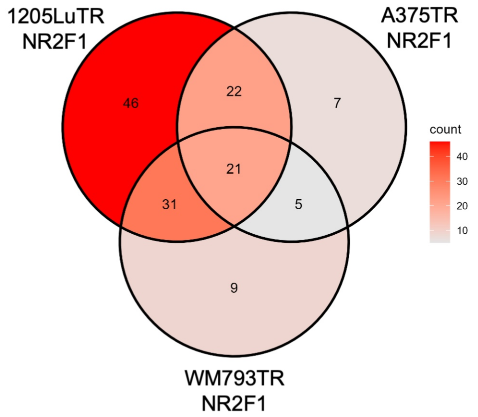
1205LuTR
NR2F1
A375TR
NR2F1
WM793TR
NR2F1
46
22
7
21
31
5
9
count
40
30
20
10

A

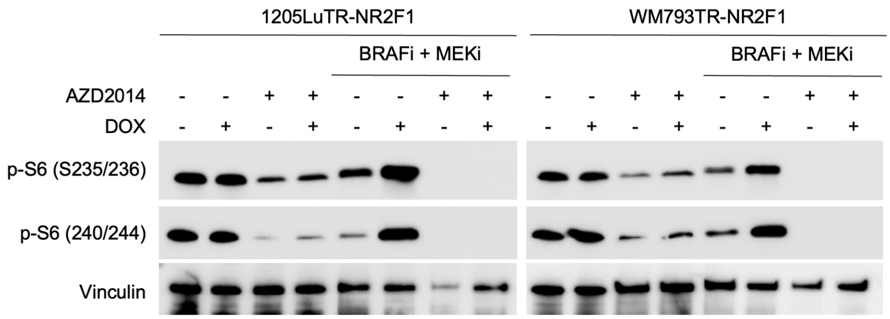
1205LuTR-NR2F1
WM793TR-NR2F1
BRAFi + MEKi
BRAFi + MEKi
AZD2014
DOX
p-S6 (S235/236)
p-S6 (240/244)
Vinculin

B

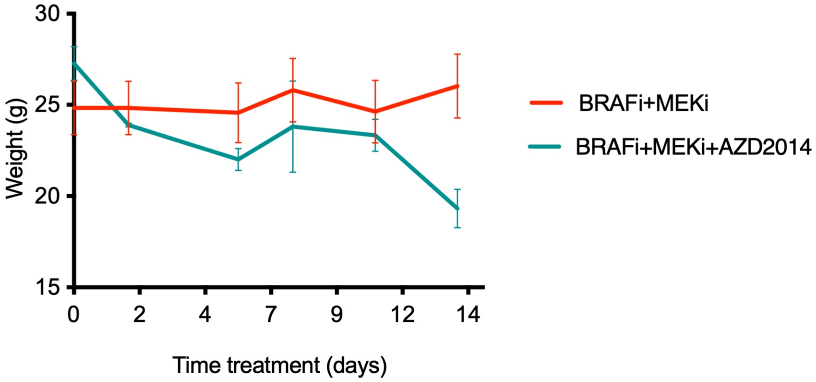
Weight (g)
30
25
20
15
0
2
4
7
9
12
14
Time treatment (days)
BRAFi+MEKi
BRAFi+MEKi+AZD2014

C

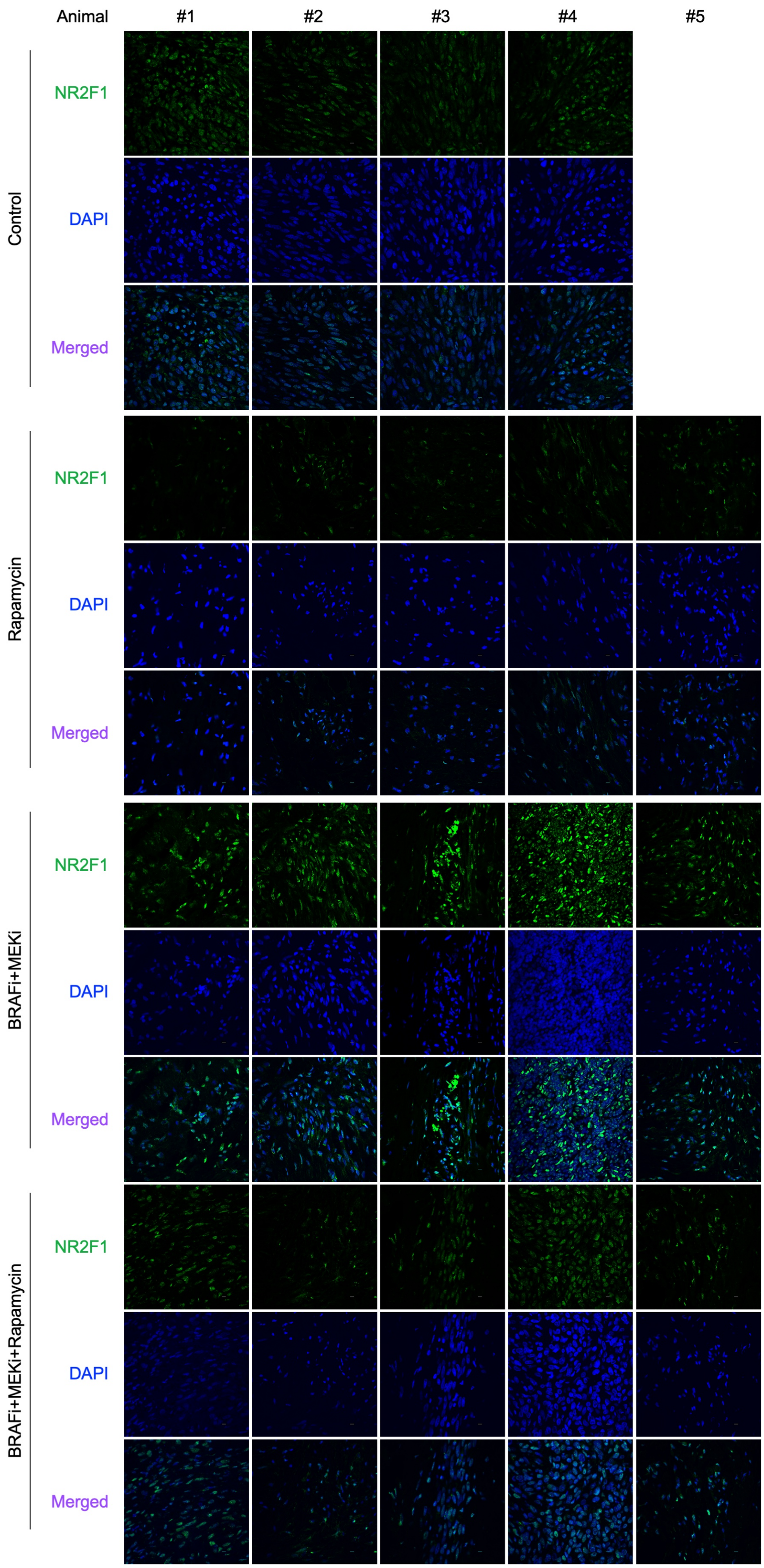
Animal
#1
#2
#3
#4
#5
Control
NR2F1
DAPI
Merged
Rapamycin
NR2F1
DAPI
Merged
BRAFi+MEKi
NR2F1
DAPI
Merged
BRAFi+MEKi+Rapamycin
NR2F1
DAPI
Merged

D

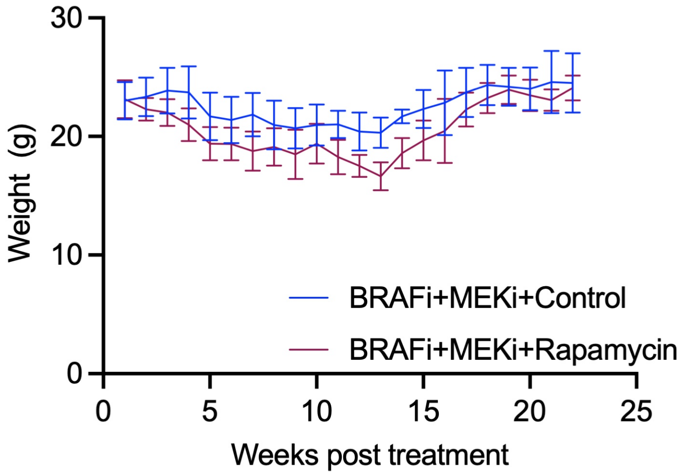
30
20
10
0
Weight (g)
0
5
10
15
20
25
Weeks post treatment
BRAFi+MEKi+Control
BRAFi+MEKi+Rapamycin

A

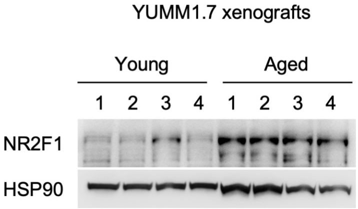
YUMM1.7 xenografts
Young
Aged
1 2 3 4 1 2 3 4
NR2F1
HSP90

B

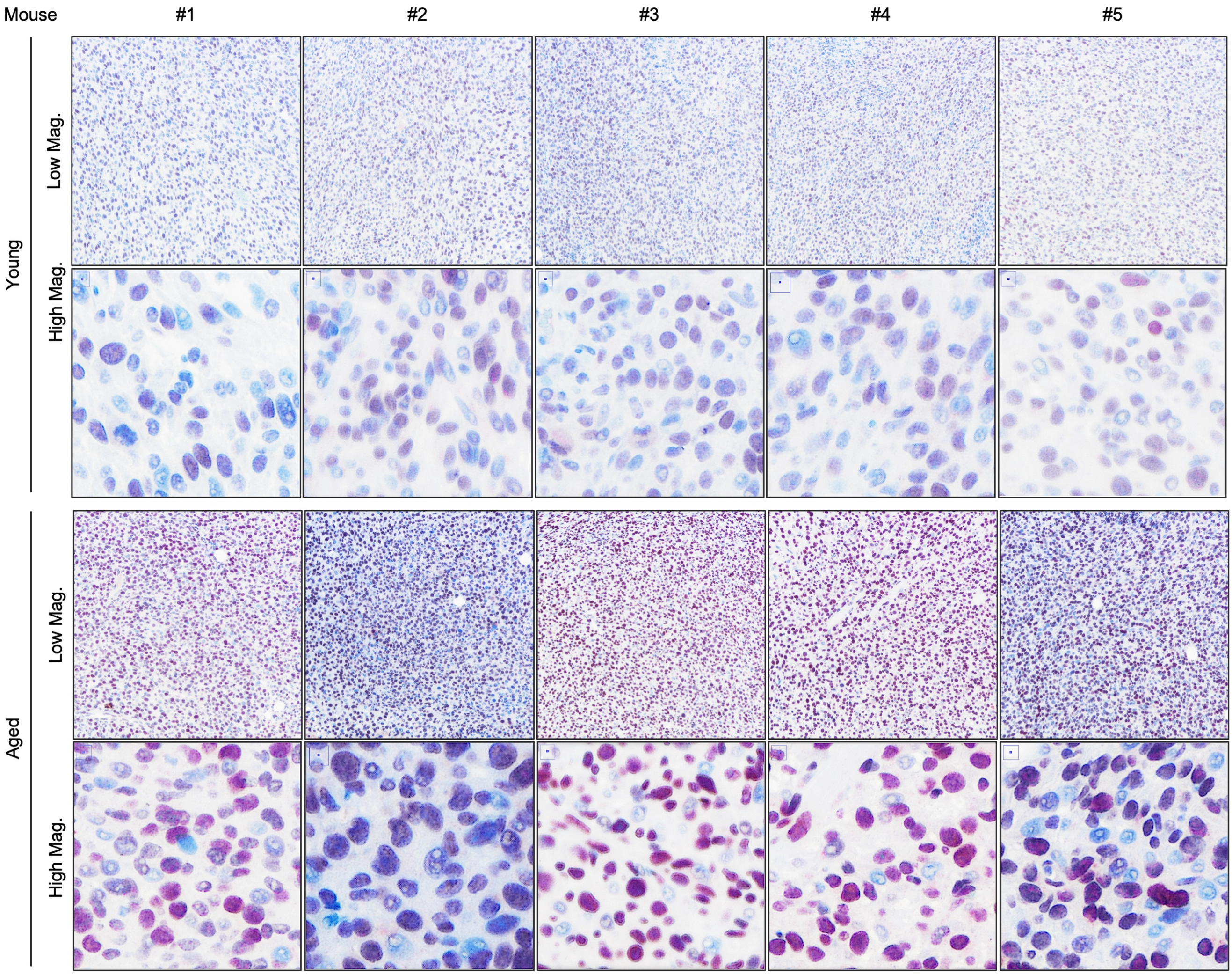
Mouse
#1
#2
#3
#4
#5
Young
Low Mag.
High Mag.
Aged
Low Mag.
High Mag.

Chemical structures for agents used in this manuscript:

PLX4720

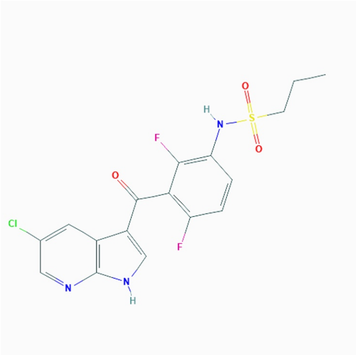

Molecular Formula:
$C_{17}H_{14}ClF_2N_3O_3S$

PD0325901

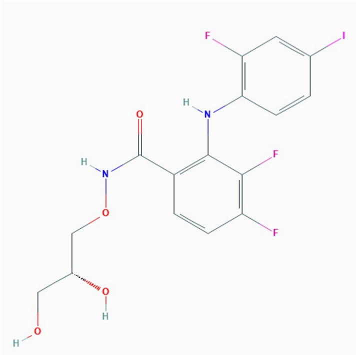

Molecular Formula:
$C_{16}H_{14}F_3IN_2O_4$

Doxycycline Hyclate

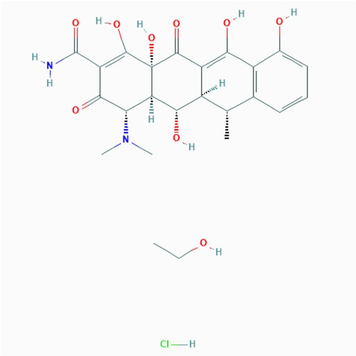

Molecular Formula:
$C_{24}H_{31}ClN_2O_9$

PD0332991

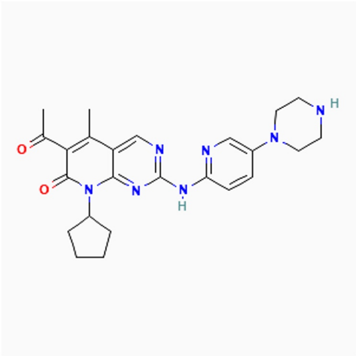

Molecular Formula:
$C_{24}H_{29}N_7O_9$

Source: https://pubchem.ncbi.nlm.nih.gov/
