# Supplementary material for: Elevated NR2F1 underlies the persistence of invasive disease after treatment of BRAF-mutant melanoma
Source: J Clin Invest. 2025 Jul 29;135(18):e178446. doi: 10.1172/JCI178446 (PMC12435839; doi:10.1172/JCI178446)

Western blot (whole blots):

A

Figure 2A:

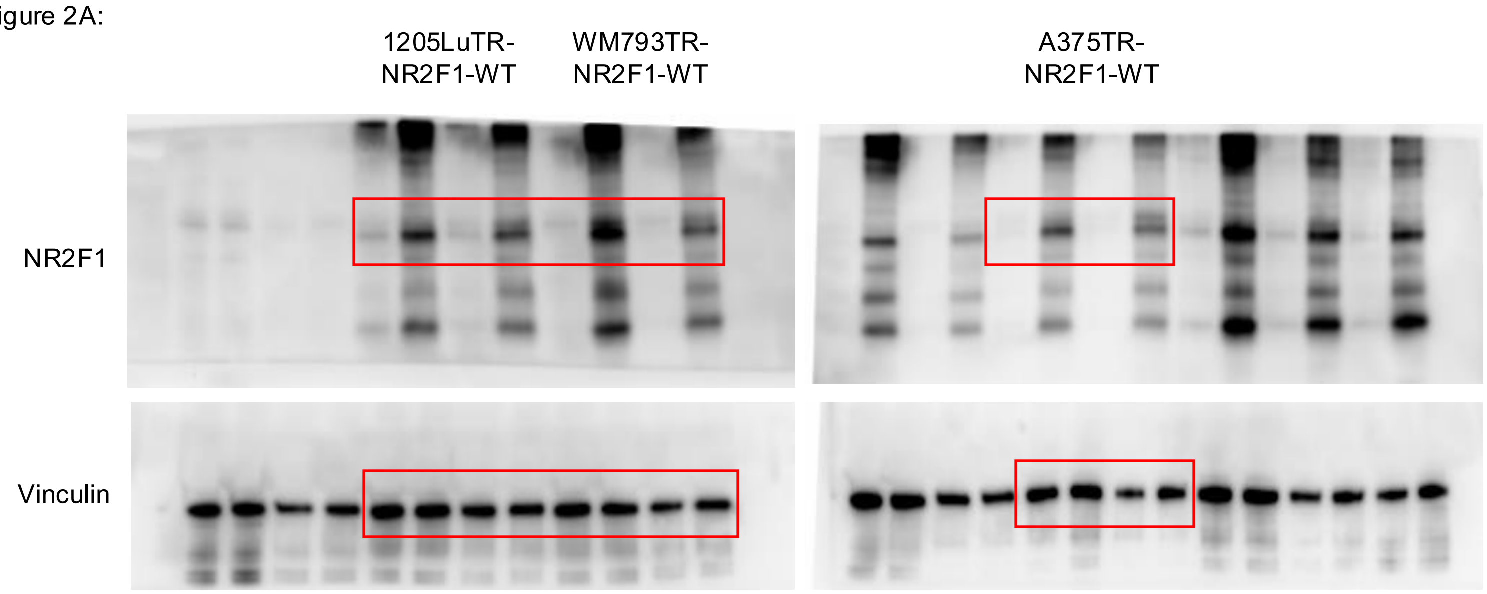

B

Figure 3B:

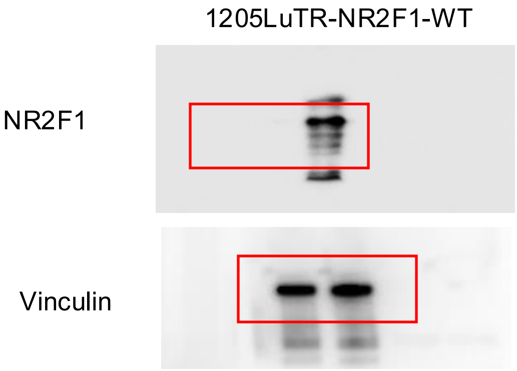

C

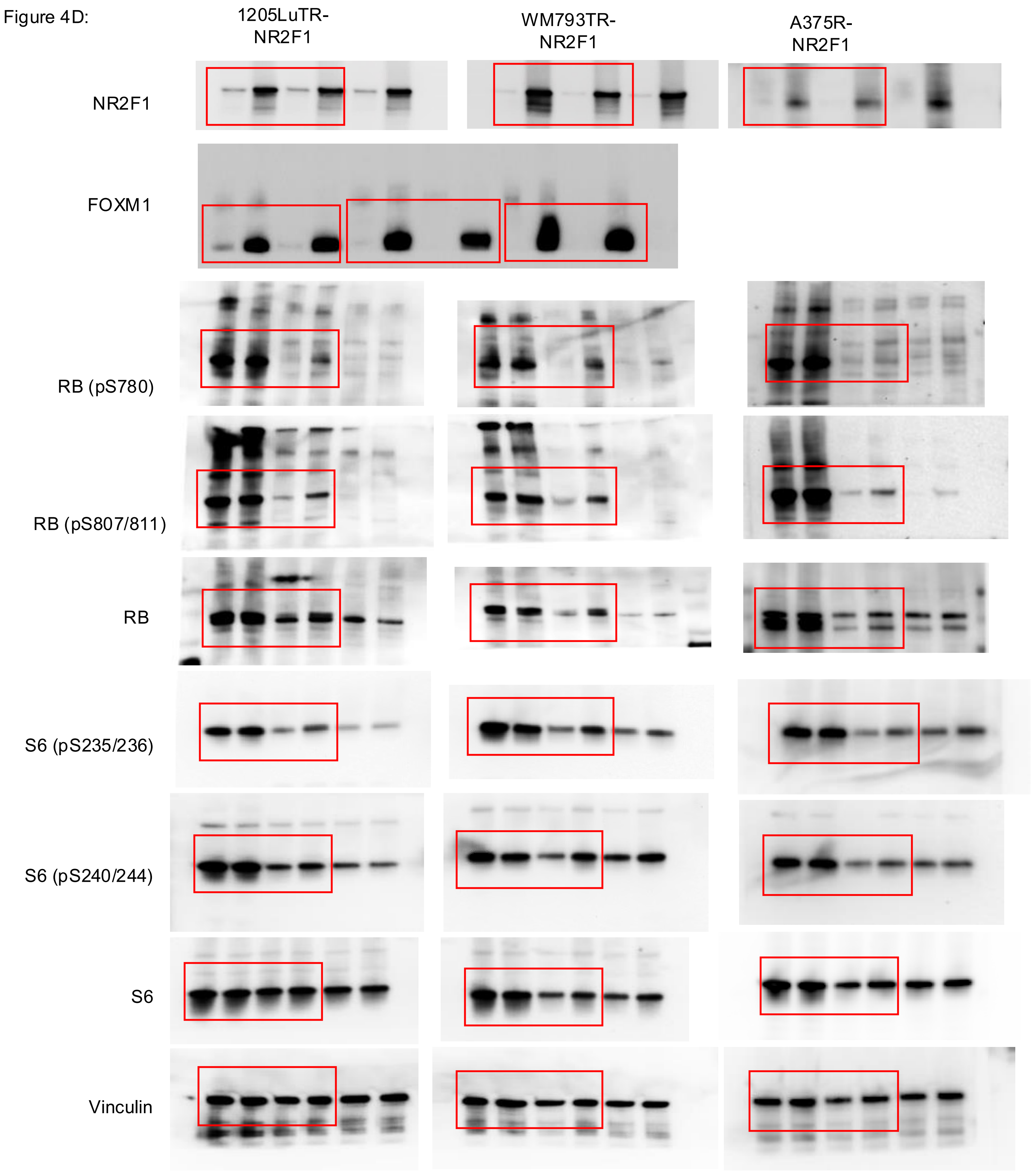
Figure 4D:
1205LuTR-NR2F1
WM793TR-NR2F1
A375R-NR2F1
NR2F1
FOXM1
RB (pS780)
RB (pS807/811)
RB
S6 (pS235/236)
S6 (pS240/244)
S6
Vinculin

D

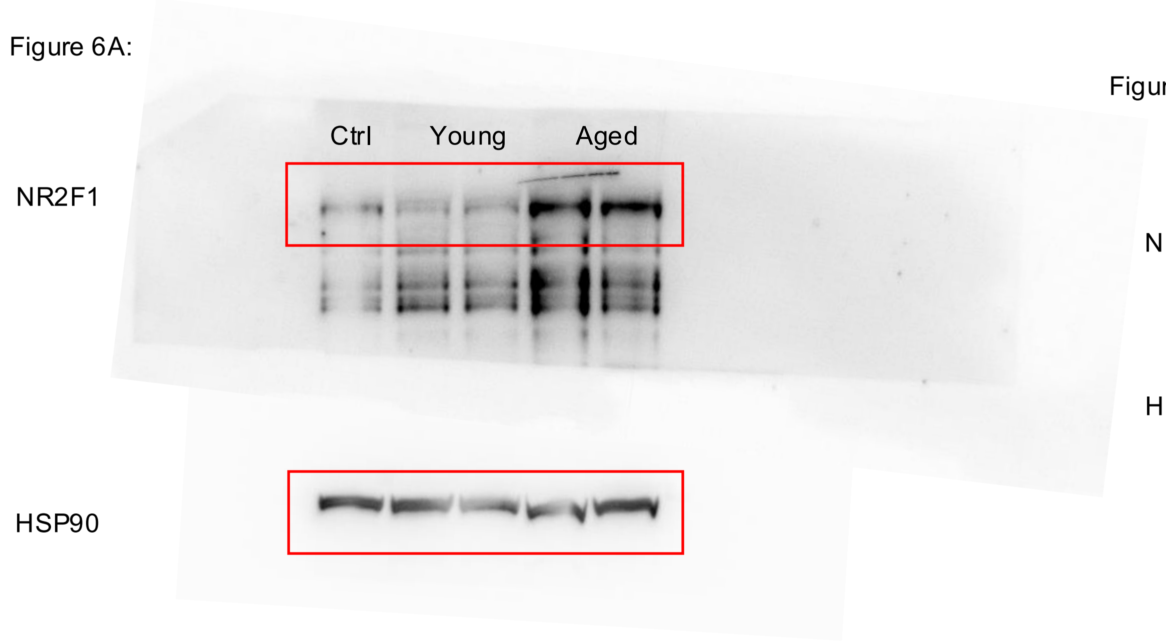
Figure 6A:
Ctrl
Young
Aged
NR2F1
HSP90

E

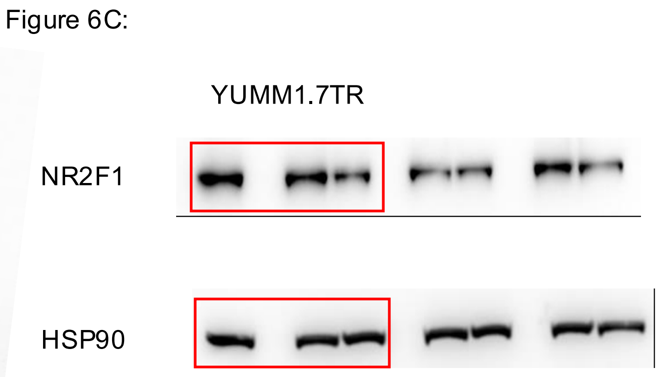
Figure 6C:
YUMM1.7TR
NR2F1
HSP90

F Supplemental Figure S1G:

NR2F1

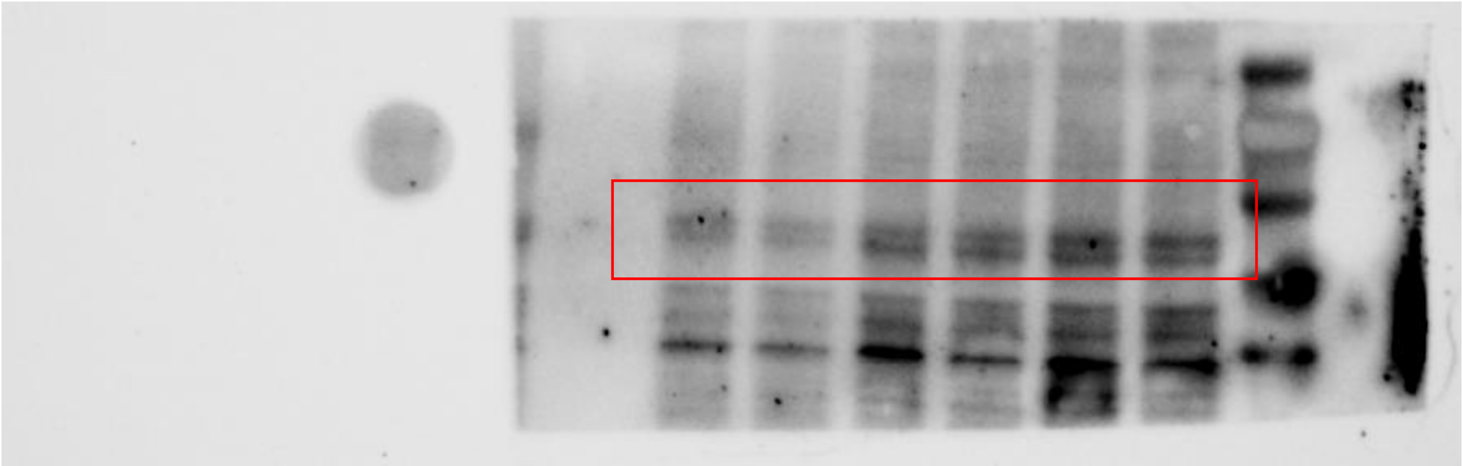

HSP90

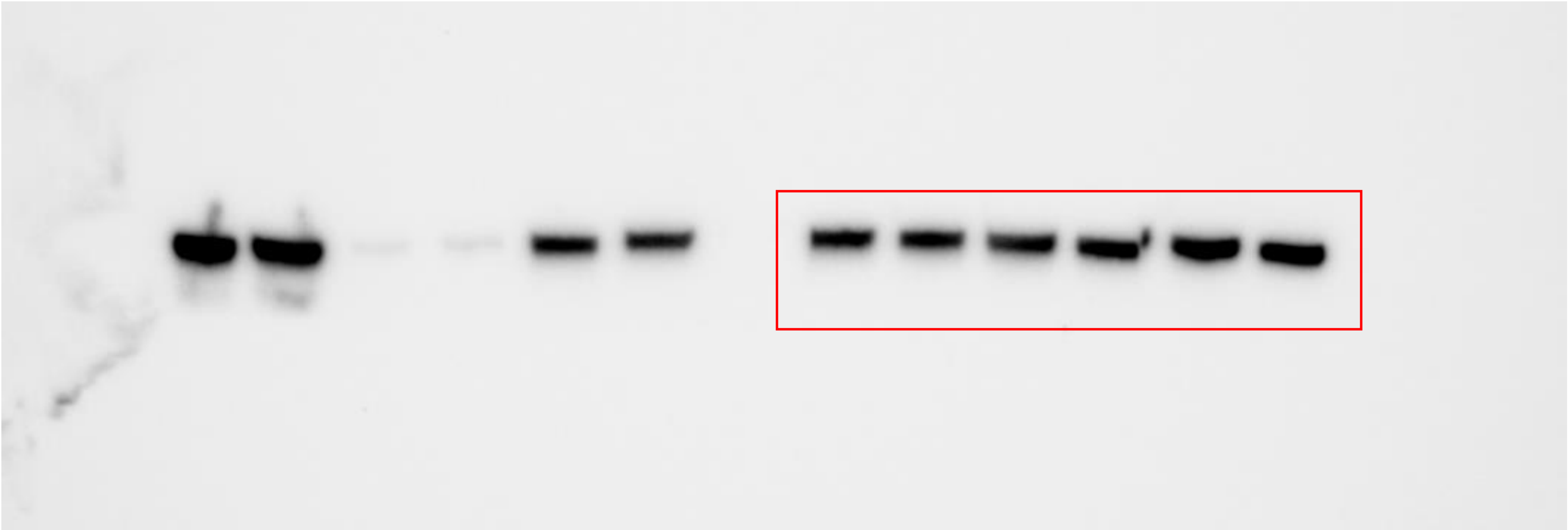

G

Supplemental Figure S2A:

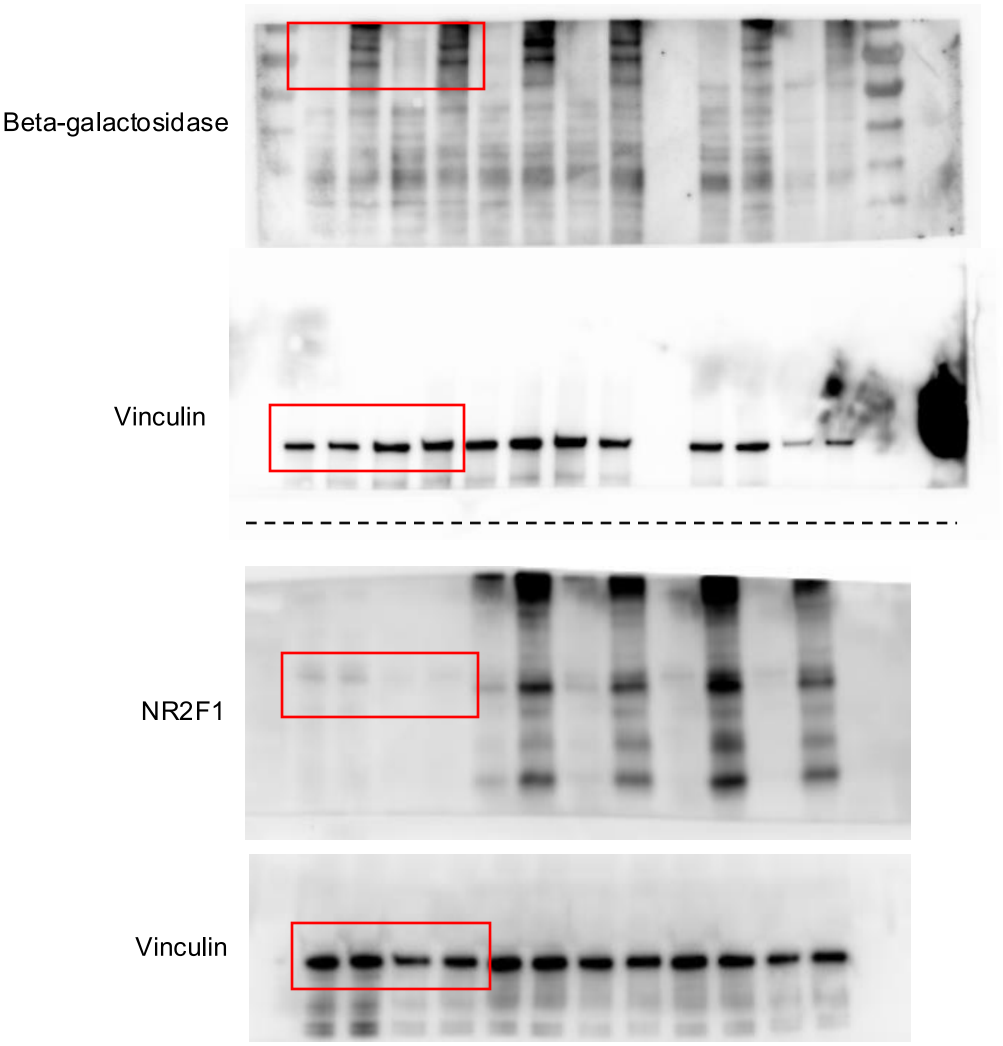

H

Supplemental Figure S4A:

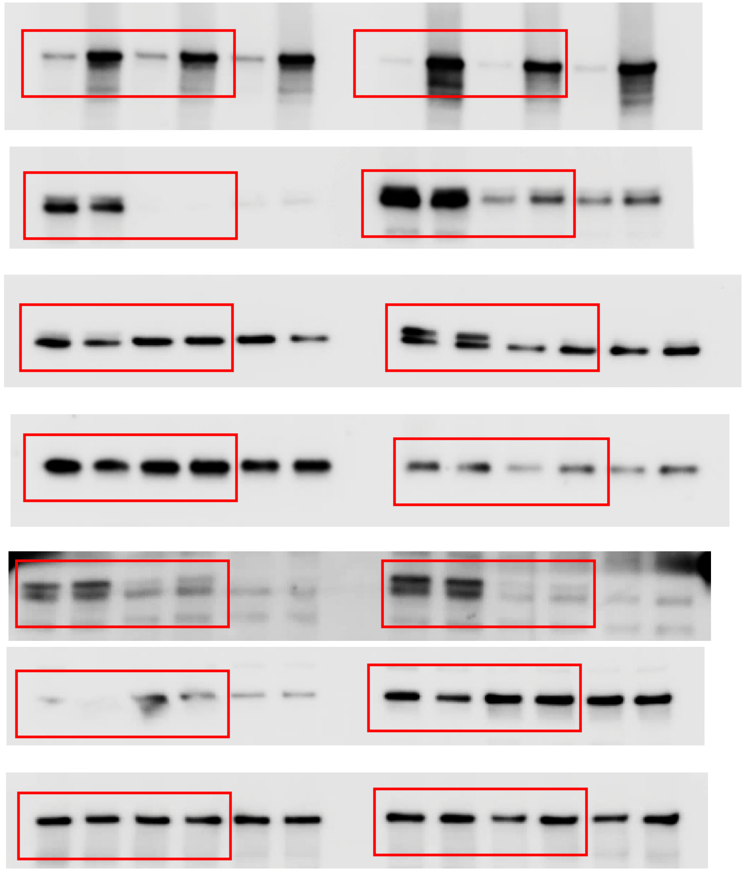

I

Supplemental Figure S4B:

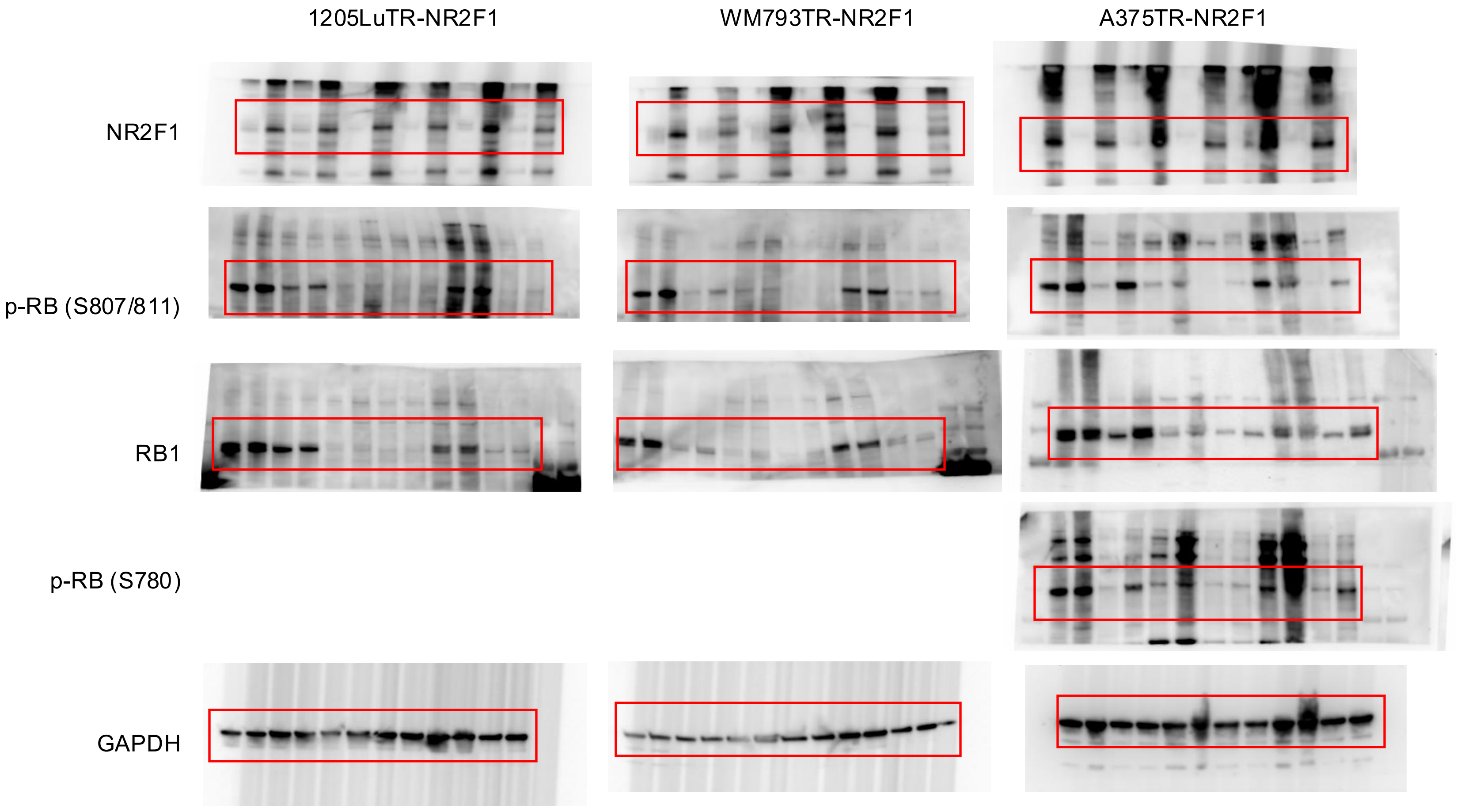

J

Supplemental Figure S4C:

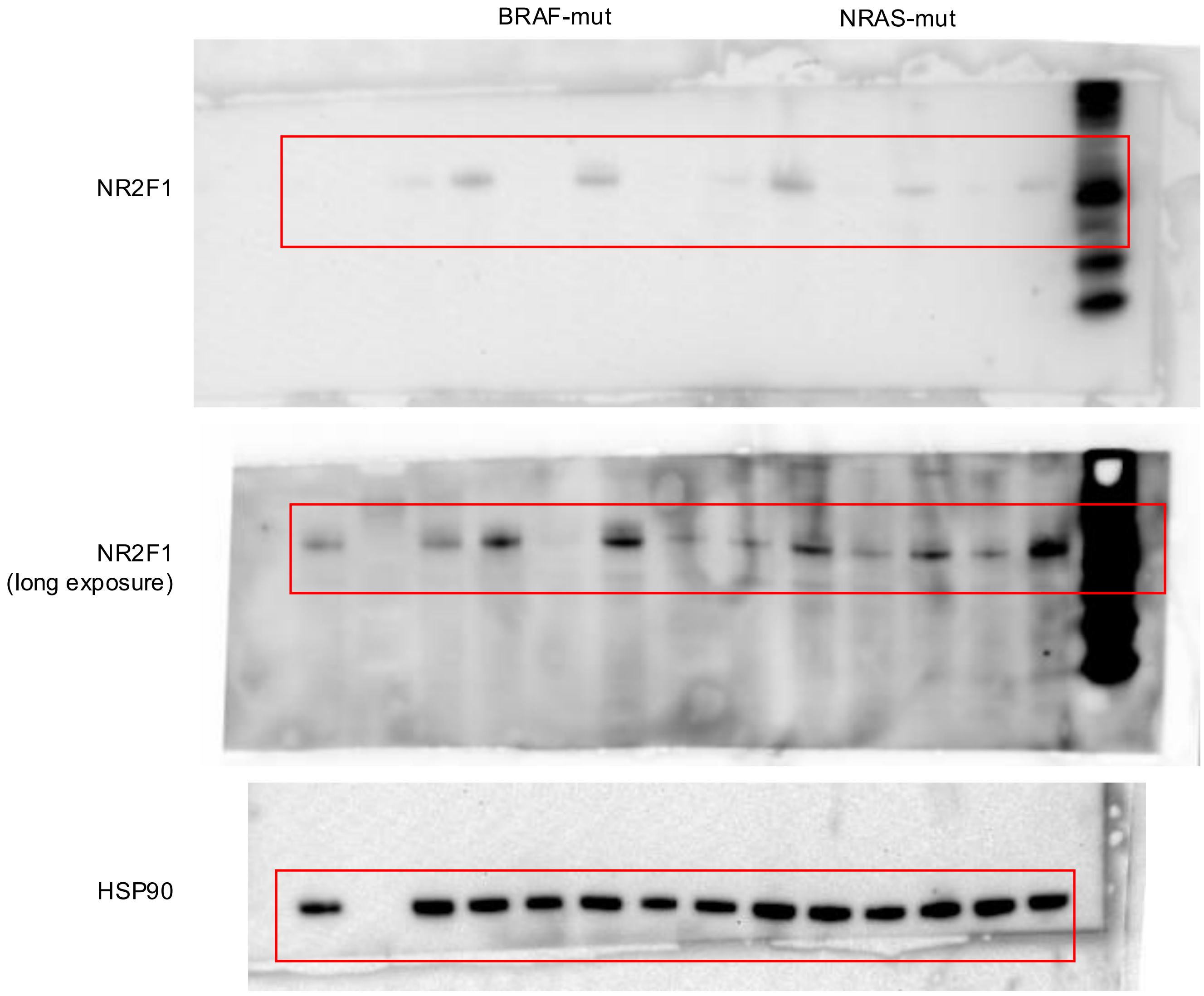

K

Supplemental Figure S5A:

1205LuTR-NR2F1

WM793TR-NR2F1

p-S6 (S235/236)

p-S6 (240/244)

Vinculin

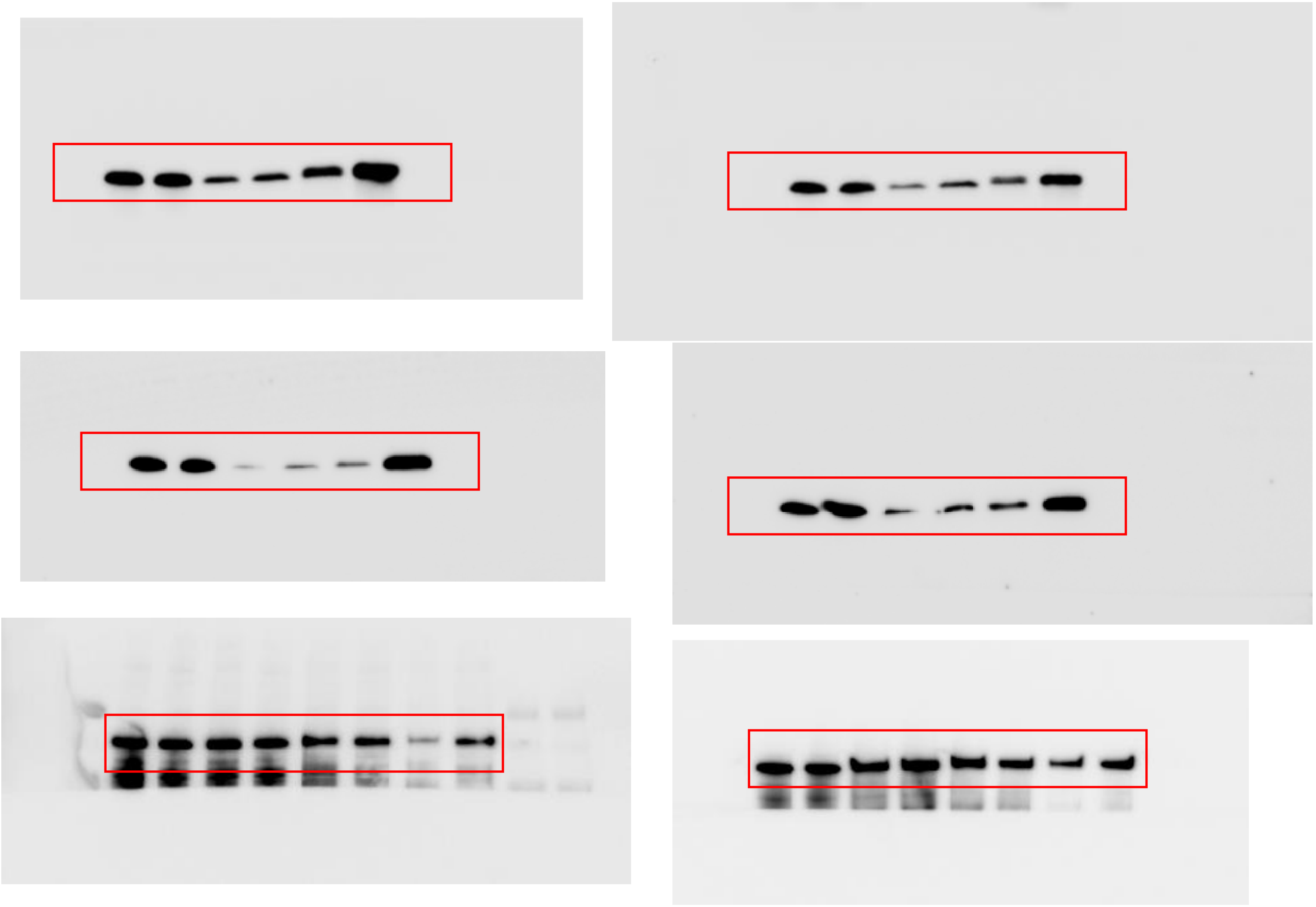

L

Supplemental Figure S6A:

YUMM1.7 xenografts

NR2F1

HSP90

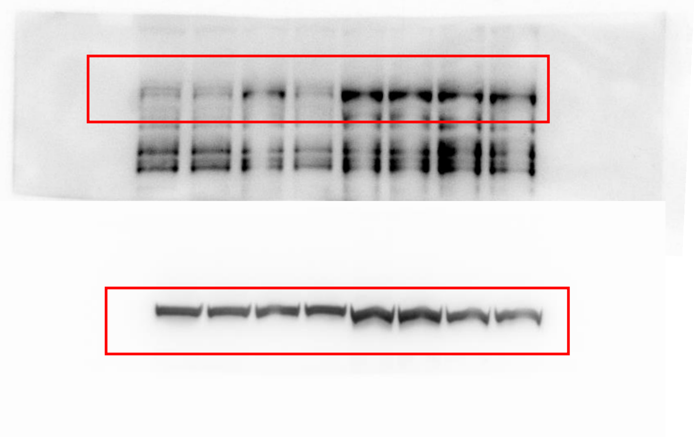

Supplement: Unedited blot and gel images [file jci-135-178446-s191.pdf]
